# *Neisseria gonorrhoeae* employs two protein inhibitors to evade killing by human lysozyme

**DOI:** 10.1371/journal.ppat.1007080

**Published:** 2018-07-05

**Authors:** Stephanie A. Ragland, Marίa V. Humbert, Myron Christodoulides, Alison K. Criss

**Affiliations:** 1 Department of Microbiology, Immunology, and Cancer Biology, University of Virginia, Charlottesville, Virginia, United States of America; 2 Neisseria Research, Molecular Microbiology, Academic Unit of Clinical and Experimental Sciences, Sir Henry Wellcome Laboratories, University of Southampton Faculty of Medicine, Southampton, United Kingdom; University of Oxford, UNITED KINGDOM

## Abstract

The bacterial pathogen *Neisseria gonorrhoeae* (Gc) infects mucosal sites rich in antimicrobial proteins, including the bacterial cell wall-degrading enzyme lysozyme. Certain Gram-negative bacteria produce protein inhibitors that bind to and inhibit lysozyme. Here, we identify Ng_1063 as a new inhibitor of lysozyme in Gc, and we define its functions in light of a second, recently identified lysozyme inhibitor, Ng_1981. *In silico* analyses indicated that Ng_1063 bears sequence and structural homology to MliC-type inhibitors of lysozyme. Recombinant Ng_1063 inhibited lysozyme-mediated killing of a susceptible mutant of Gc and the lysozyme-sensitive bacterium *Micrococcus luteus*. This inhibitory activity was dependent on serine 83 and lysine 103 of Ng_1063, which are predicted to interact with lysozyme’s active site residues. Lysozyme co-immunoprecipitated with Ng_1063 and Ng_1981 from intact Gc. Ng_1063 and Ng_1981 protein levels were also increased in Gc exposed to lysozyme. Gc lacking both *ng1063* and *ng1981* was significantly more sensitive to killing by lysozyme than wild-type or single mutant bacteria. When exposed to human tears or saliva, in which lysozyme is abundant, survival of *Δ1981Δ1063* Gc was significantly reduced compared to wild-type, and survival was restored upon addition of recombinant Ng_1981. *Δ1981Δ1063* mutant Gc survival was additionally reduced in the presence of human neutrophils, which produce lysozyme. We found that while Ng_1063 was exposed on the surface of Gc, Ng_1981 was both in an intracellular pool and extracellularly released from the bacteria, suggesting that Gc employs these two proteins at multiple spatial barriers to fully neutralize lysozyme activity. Together, these findings identify Ng_1063 and Ng_1981 as critical components for Gc defense against lysozyme. These proteins may be attractive targets for antimicrobial therapy aimed to render Gc susceptible to host defenses and/or for vaccine development, both of which are urgently needed against drug-resistant gonorrhea.

## Author summary

The mucosal pathogen *Neisseria gonorrhoeae* has acquired resistance to almost all recommended antibiotics, and no gonorrhea vaccine currently exists. Attractive targets for therapeutic discovery include bacterial factors that, when inactivated, enhance bacterial susceptibility to host-derived antimicrobial components. The bacterial cell wall-degrading enzyme lysozyme is abundant in mucosal secretions and innate immune cells. To resist killing by lysozyme, some bacteria produce proteins that bind to and directly inhibit the activity of lysozyme. Here, we demonstrate lysozyme inhibitory activity in the *N*. *gonorrhoeae* protein Ng_1063. We found that both Ng_1063 and a second, recently described lysozyme inhibitor, Ng_1981, contribute to full resistance of *N*. *gonorrhoeae* to lysozyme, including resistance to lysozyme-rich mucosal secretions and human neutrophils. Although Ng_1063 and Ng_1981 are both inhibitors of lysozyme, they are distinct in their sequences, biological activities, and cellular localizations. Because both Ng_1063 and Ng_1981 are extracellular, we propose they can be targeted for vaccines and drugs that sensitize Gc to human antimicrobial defenses.

## Introduction

*Neisseria gonorrhoeae* (Gc) is a Gram-negative diplococcus and the causative agent of the sexually transmitted infection gonorrhea. The World Health Organization (WHO) estimates 78 million cases of gonorrhea occur each year, with over 800,000 cases reported annually in the United States [1–3]. The lack of a protective vaccine, widespread prevalence of antibiotic-resistant Gc, and treatment failures with last line therapeutics have prompted the United States Centers for Disease Control to label antibiotic-resistant Gc as an urgent threat to public health [1, 4–6]. Likewise, the WHO lists the control and elimination of Gc infection as a high priority [7]. Dissecting Gc pathogenesis and virulence is critical for the development of novel therapeutics and vaccines.

Gc colonizes mucosal sites, including the cervix, urethra, pharynx, conjunctiva, and rectum. Colonization initiates an inflammatory response, culminating in the robust recruitment of neutrophils, an innate immune cell with antimicrobial killing activities [8]. Thus, Gc survival during human infection requires defenses against the antimicrobial molecules made both by the mucosal epithelium and neutrophils. For instance, Gc evades killing by cationic antimicrobial proteins by modifying surface lipooligosaccharide and producing multidrug efflux pumps, and these activities are important for Gc survival from human neutrophils, survival in the mouse model of Gc cervicovaginal colonization, and/or survival in a human male urethral-infection model [9–11].

The antimicrobial protein lysozyme is ubiquitous at the mucosal sites colonized by Gc, reaching concentrations as high as 2 mg/mL in the conjunctiva and 1 mg/mL in the cervix [12, 13]. Lysozyme is also abundant in phagocytes like neutrophils [14, 15]. Lysozyme hydrolyzes the glycan backbone of bacterial cell wall peptidoglycan, causing lysis and death. Because lysozyme is highly cationic, it can also kill bacteria through an enzymatic-independent mechanism, purportedly via pore formation on bacterial membranes [16–19]. Bacteria have evolved numerous, non-redundant mechanisms to thwart the killing activities of lysozyme, which in many cases contribute to enhanced survival and virulence *in vivo* (reviewed in [20]). These observations highlight lysozyme as a critical player in host defense, and, in turn, underscore the importance for lysozyme resistance to a pathogen’s success.

Lysozyme resistance in Gc is mediated by envelope integrity, peptidoglycan modifications, and protein inhibitors of lysozyme. In Gram-negative bacteria like Gc, the outer membrane (OM) restricts periplasmic access of lysozyme [21]. Consequently, maintenance of envelope integrity is vital for Gram-negative resistance to lysozyme. We recently reported that two cell wall turnover proteins, the lytic transglycosylases LtgA and LtgD, contribute to envelope integrity in Gc, and *ΔltgAΔltgD* mutant Gc is more sensitive to killing by lysozyme and human neutrophils [22]. Another mechanism of lysozyme resistance in Gc involves *O*-acetylation of peptidoglycan, which can sterically hinder lysozyme’s hydrolytic activity [23, 24]. *O*-acetylation, however, only contributes to lysozyme resistance in Gc if the envelope is also compromised, as when LtgA and LtgD are lost [22, 24].

Recently, we identified a third mechanism of lysozyme resistance in *Neisseria*, the production of a protein inhibitor of lysozyme [25]. Protein inhibitors have only been identified in Gram-negative bacteria, and their expression contributes to bacterial survival within mucosal secretions, survival from phagocytes, and/or survival *in vivo* [26–30]. Inhibitors against c-type lysozymes, like human lysozyme, are classified as Ivy (inhibitor of vertebrate lysozyme), PliC (periplasmic lysozyme inhibitor of c-type lysozyme), or MliC (membrane-bound lysozyme inhibitor of c-type lysozyme). PliC- and MliC-type inhibitors share structural similarity and conserved sequence motifs that are distinct from Ivy-type inhibitors [31]. All function by insertion of one or more protein loops into the active site of lysozyme to interfere with its peptidoglycan-hydrolyzing activity [31]. We found that purified recombinant Ng_1981 (also known as the Ng-Adhesin Complex Protein, Ng-ACP) from Gc inhibits the enzymatic activity of lysozyme *in vitro* and contributes to gonococcal tolerance to lysozyme [25]. The homolog of Ng_1981 in *Neisseria meningitidis*, NMB_2095 (Nm-ACP), is 94% identical to Ng_1981 and binds directly to lysozyme with micromolar affinity [25]. Although the structure of NMB_2095 exhibits overall similarity with PliC/MliC-type inhibitors, NMB_2095 and Ng_1981 lack the conserved PliC/MliC sequence features and therefore were classified as a novel type of lysozyme inhibitor [25, 31].

Gram-negative bacteria often produce multiple, non-redundant inhibitors of lysozyme [26–28, 32–35], prompting us to investigate whether Gc also produces more than one inhibitor. In this work, we report that the open reading frame *ng1063* (KEGG GENOME, Gc strain FA1090) encodes for a protein that shares sequence and structural homology with MliC-type inhibitors. We tested the hypothesis that Ng_1063 functions as a lysozyme inhibitor in Gc and further defined its biological activity in the context of Ng_1981. Our findings suggest that Ng_1063 functions as a bona fide inhibitor of lysozyme and that Gc employs both Ng_1063 and Ng_1981, which exhibit distinct properties and localizations, for optimal defense against lysozyme.

## Results

### Ng_1063 is a protein inhibitor of lysozyme

**Fig 1:**
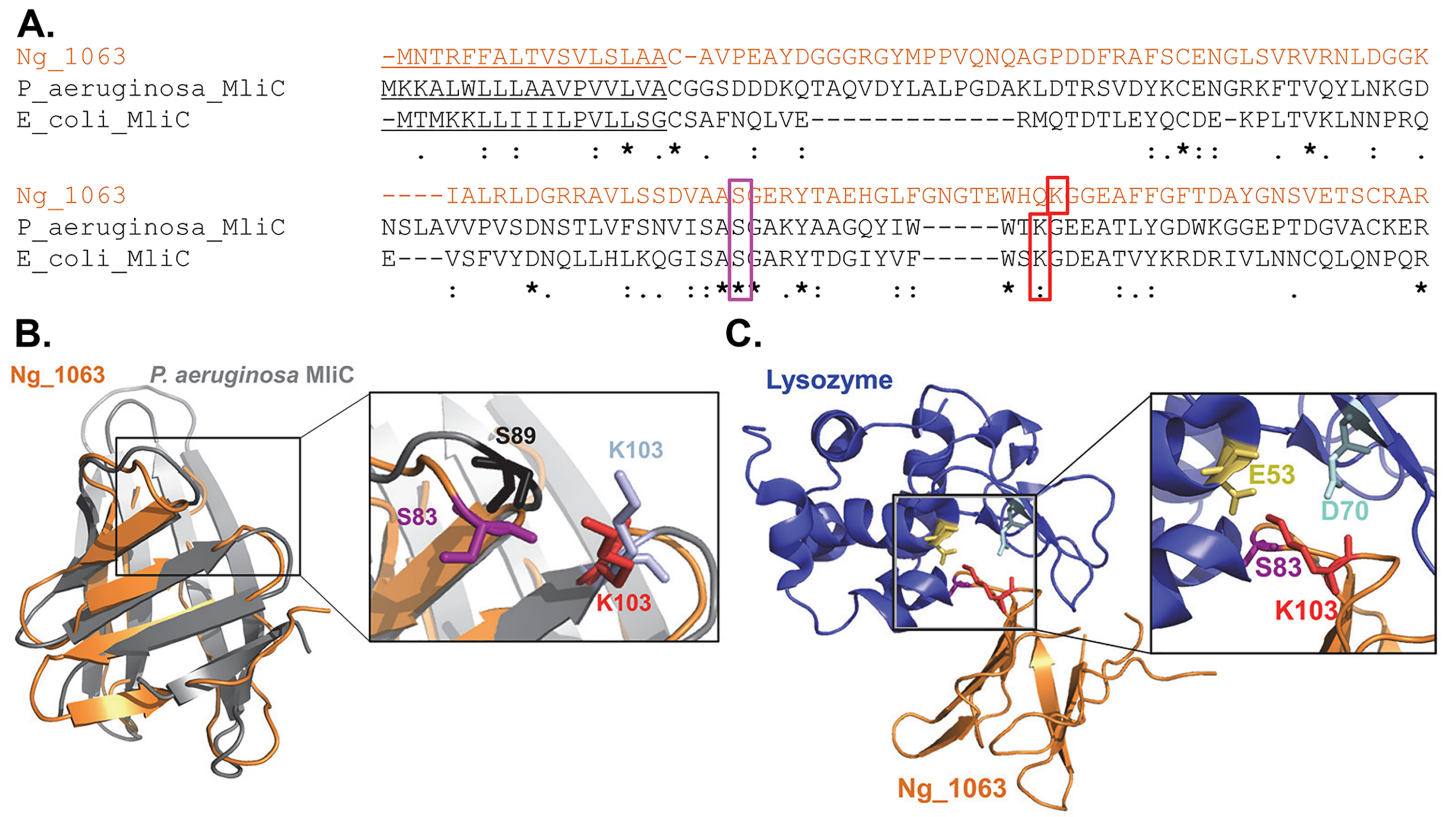
*In silico* analysis of Ng_1063. A. MUSCLE alignment of Ng_1063 (strain MS11, orange text) with *P*. *aeruginosa* and *E*. *coli* MliC proteins. Signal sequence is underlined. Asterisks (*) denote positions in the sequence with a fully conserved residue. Colons (:) and periods (.) denote amino acids with strongly or weakly similar properties, respectively. The serine and lysine residues implicated in MliC inhibition of lysozyme are highlighted in magenta and red boxes, respectively. B. Alignment of the predicted structure of Ng_1063 (orange) with *P*. *aeruginosa* MliC (grey). The structure of Ng_1063 (strain MS11) was predicted using the PHYRE^2^ server and subsequently aligned to *P*. *aeruginosa* MliC (PDB 3f6z) via PyMOL. Inset shows the S83 (magenta) and K103 (red) residues from Ng_1063 and the S89 (black) and K103 (light blue) residues from *P*. *aeruginosa* MliC. C. Predicted Ng_1063 (orange) complex with lysozyme (hen egg white, blue). The *P*. *aeruginosa* MliC complex with lysozyme (PDB 3f6z) was used as a platform to model the Ng_1063-lysozyme complex via PyMOL. Inset shows the S83 (magenta) and K103 (red) residues from Ng_1063 with the lysozyme active site residues, E53 (yellow) and D70 (cyan).

Ng_1063 shares overall amino acid sequence similarity with MliC from *P*. *aeruginosa* (23% identity, 40% similarity) and *E*. *coli* (16% identity, 32% similarity) (Fig 1A). S89 and K103 of *P*. *aeruginosa* MliC interact with the active site residues of lysozyme and are required for MliC inhibitory function [36]; the corresponding residues, S83 and K103, are conserved in Ng_1063 (Fig 1A). Like other MliC inhibitors, Ng_1063 is predicted to be a lipoprotein as determined by signal sequence analysis using lipoP 1.0 (http://www.cbs.dtu.dk/services/LipoP/). We characterized the potential for Ng_1063 as an MliC-type inhibitor of lysozyme using PHYRE^2^ (www.sbg.bio.ic.ac.uk/phyre2) to predict the Ng_1063 three-dimensional structure. PHYRE^2^ predicted Ng_1063 to have an MliC-type fold with 99.6% confidence. Alignment of the Ng_1063 predicted structure with *P*. *aeruginosa* MliC in PyMOL shows overlap of S83 and K103 in Ng_1063 with the corresponding residues of *P*. *aeruginosa* MliC (Fig 1B). Threading of Ng_1063 through the *P*. *aeruginosa* MliC-lysozyme co-crystal structure (PDB 3f6z) positions the S83 and K103 residues of Ng_1063 in close proximity to the active site residues of lysozyme, E53 and D70 (Fig 1C). Based on these *in silico* analyses, we hypothesized that Ng_1063 is a protein inhibitor of lysozyme.

**Fig 2:**
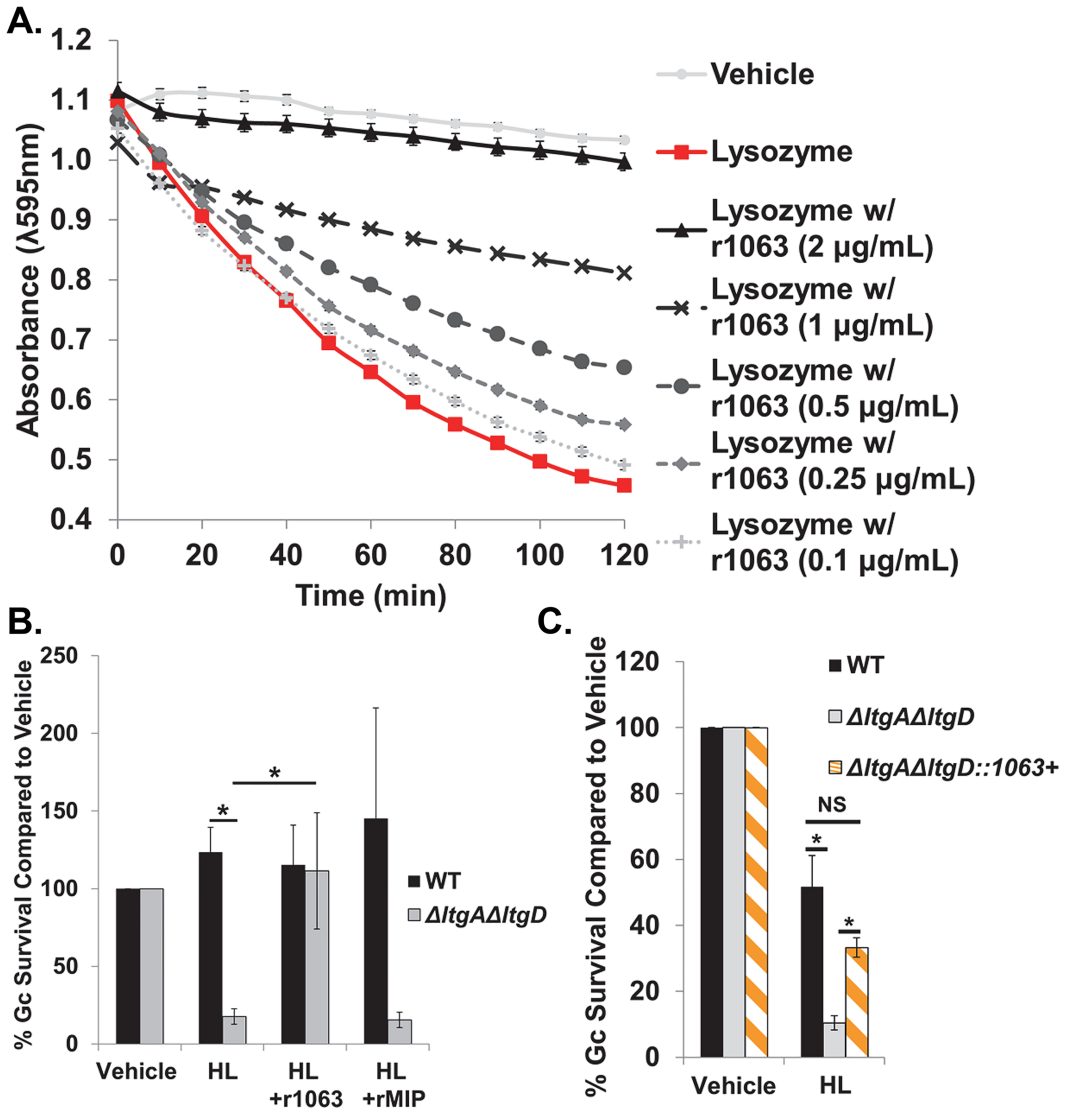
Ng_1063 inhibits human lysozyme killing activities *in vitro*. A. *M*. *luteus* was exposed to vehicle control, 2 μg/mL human lysozyme alone, or 2 μg/mL human lysozyme with increasing concentrations of recombinant 1063 protein (r1063 concentrations indicated in parentheses). Loss of absorbance at λ595 nm, indicating bacterial lysis, was measured over time. *n* = 3 biological replicates. Differences between vehicle control and lysozyme with 2 μg/mL r1063 were not significant at any time point. B. MS11 WT and *ΔltgAΔltgD* Gc were exposed to 1.5 μg/mL human lysozyme (HL), which had been left untreated or pretreated with r1063 (0.75 μg/mL final in assay) or recombinant MIP (rMIP, 0.75 μg/mL final in assay), for 5hr. Percent Gc survival was determined by dividing the CFU/mL at 5 hr by the CFU/mL at 0 hr and normalized to the vehicle control (100%). *n* = 6–15 biological replicates. C. WT, *ΔltgAΔltgD*, and *ΔltgAΔltgD*::*1063+* complement Gc were exposed to 10 μg/mL human lysozyme (HL) for 3 hr. Gc survival was determined as in Fig. 2B. NS, not significant. *n* = 3–6 biological replicates. All values are represented as the mean ± SEM. *p < 0.05; two tailed *t*-test.

To test this hypothesis, we first examined if Ng_1063 protein could rescue the lysozyme-mediated lysis of *Micrococcus luteus*, a Gram-positive bacterium that is intrinsically sensitive to lysozyme. We generated purified, recombinant Ng_1063 (r1063) protein (see Materials and methods) and found it prevented the lytic activity of lysozyme on *M*. *luteus* in a concentration-dependent manner (Fig 2A). As Gc is the relevant context for Ng_1063 activity, we next investigated the effect of r1063 on survival of Gc after exposure to lysozyme. In contrast to *M*. *luteus*, WT Gc is relatively resistant to lysozyme. Thus, we used an *ΔltgAΔltgD* Gc mutant, which we previously demonstrated has increased sensitivity to lysozyme, as a tool to assess r1063 inhibitory activity with Gc [22]. As expected, *ΔltgAΔltgD* mutant Gc was markedly reduced in survival in the presence of lysozyme, compared to WT (strain MS11) (Fig 2B). Preincubation of lysozyme with r1063, but not an unrelated *Neisserial* OM protein (macrophage infectivity potentiator (MIP) [25]), restored survival of the *ΔltgAΔltgD* mutant to WT levels (Fig 2B). Overexpression of *ng1063* in *ΔltgAΔltgD* mutant Gc was also sufficient to rescue bacterial survival to WT levels (Fig 2C). Although the *ΔltgAΔltgD* mutant has reduced envelope integrity that correlates with lysozyme sensitivity [22], overexpression of *ng1063* in this mutant did not affect bacterial susceptibility to vancomycin, an indicator of cellular permeability (S1 Table). These observations strongly suggest that Ng_1063 protects Gc by inhibiting lysozyme, rather than enhancing envelope integrity.

Together, these results identify Ng_1063 as a new lysozyme inhibitor made by Gc.

### Lysozyme interacts with and induces expression of Ng_1063 and Ng_1981

Besides Ng_1063, Gc produces another inhibitor of lysozyme, Ng_1981, which we recently characterized [25]. Ng_1981 shares limited sequence similarity with Ng_1063 (19% identity, 35% similarity) (S1A Fig) yet is predicted to share a similar overall three-dimensional structure [25]. Recombinant Ng_1981 (r1981) inhibits the lytic activity of lysozyme against *M*. *luteus* [25], and we found that pretreatment of lysozyme with r1981 or overexpression of *ng1981* restored *ΔltgAΔltgD* mutant survival after exposure to lysozyme to WT levels (S1B and S1C Fig). Overexpression of *ng1981* did not affect the sensitivity of *ΔltgAΔltgD* mutant Gc to vancomycin (S1 Table).

**Fig 3:**
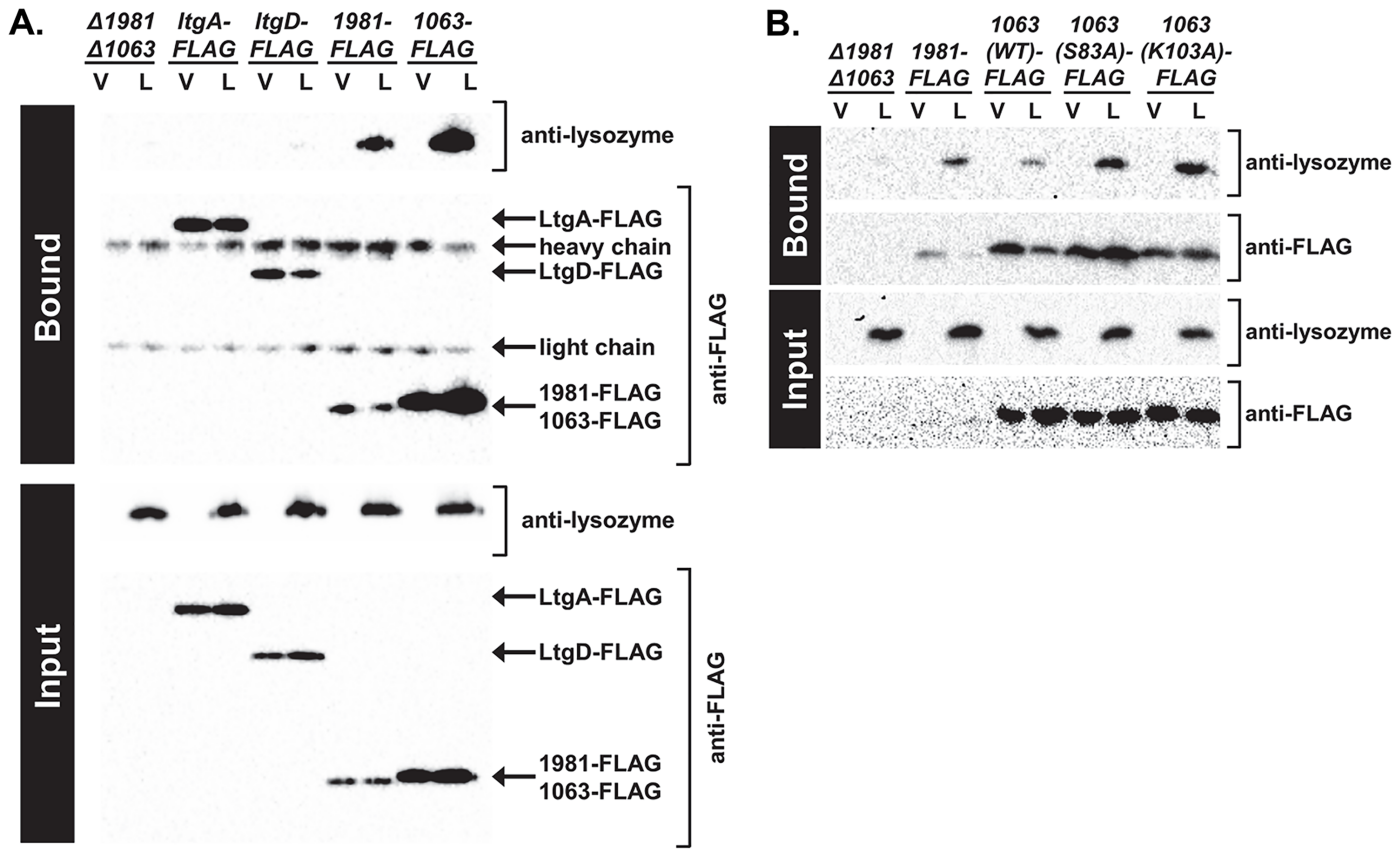
Ng_1063 and Ng_1981 interact with human lysozyme *in vivo*. A. *Δ1981Δ1063*, *ΔltgA*::*ltgA*-FLAG complement, *ΔltgD*::*ltgD*-FLAG complement, *Δ1981Δ1063*::*1981*-FLAG complement, and *Δ1981Δ1063*::*1063*(WT)-FLAG complement Gc were exposed to vehicle (V) or 1 mg/mL human lysozyme (L) for 3 hr. Gc were subsequently washed and lysed. Whole cell lysates were incubated with anti-FLAG resin for 2 hr and unbound material subsequently removed. Proteins bound to the anti-FLAG resin and whole cell lysates were analyzed by immunoblot for lysozyme and FLAG. Shown is 1 representative of 3 independent experiments. B. *Δ1981Δ1063*, *Δ1981Δ1063*::*1981*(WT)-FLAG, *Δ1981Δ1063*::*1063*(WT)-FLAG complement, *Δ1981Δ1063*::*1063*(S83A)-FLAG complement, and *Δ1981Δ1063*::*1063*(K103A)-FLAG complement Gc were exposed to vehicle (V) or human lysozyme (L) as in Fig. 3A. Shown is 1 representative of 3 independent experiments.

We next tested whether both Ng_1063 and Ng_1981 interact with lysozyme in the physiological context of the bacterial cell. We generated a *Δ1981Δ1063* mutant, where *ng1063* was replaced with a null allele and *ng1981* was disrupted by insertion-deletion. *Δ1981Δ1063* mutant Gc was then complemented with C-terminal FLAG-tagged versions of either *ng1063* or *ng1981* at an ectopic chromosomal location under IPTG regulation (see Materials and methods). As a negative control, we used Gc expressing C-terminal FLAG-tagged *ltgA* or *ltgD*, also under IPTG regulation (see Materials and methods) [37]. These strains were induced for FLAG-protein expression and incubated with lysozyme or vehicle control. FLAG-tagged proteins and their interacting partners were then immunoprecipitated from bacterial cell lysates. Lysozyme co-precipitated with Ng_1063-FLAG and Ng_1981-FLAG, but not with LtgA-FLAG, LtgD-FLAG, or anti-FLAG resin alone, showing the lysozyme inhibitors both interact with lysozyme in intact bacteria (Fig 3A).

**Fig 4:**
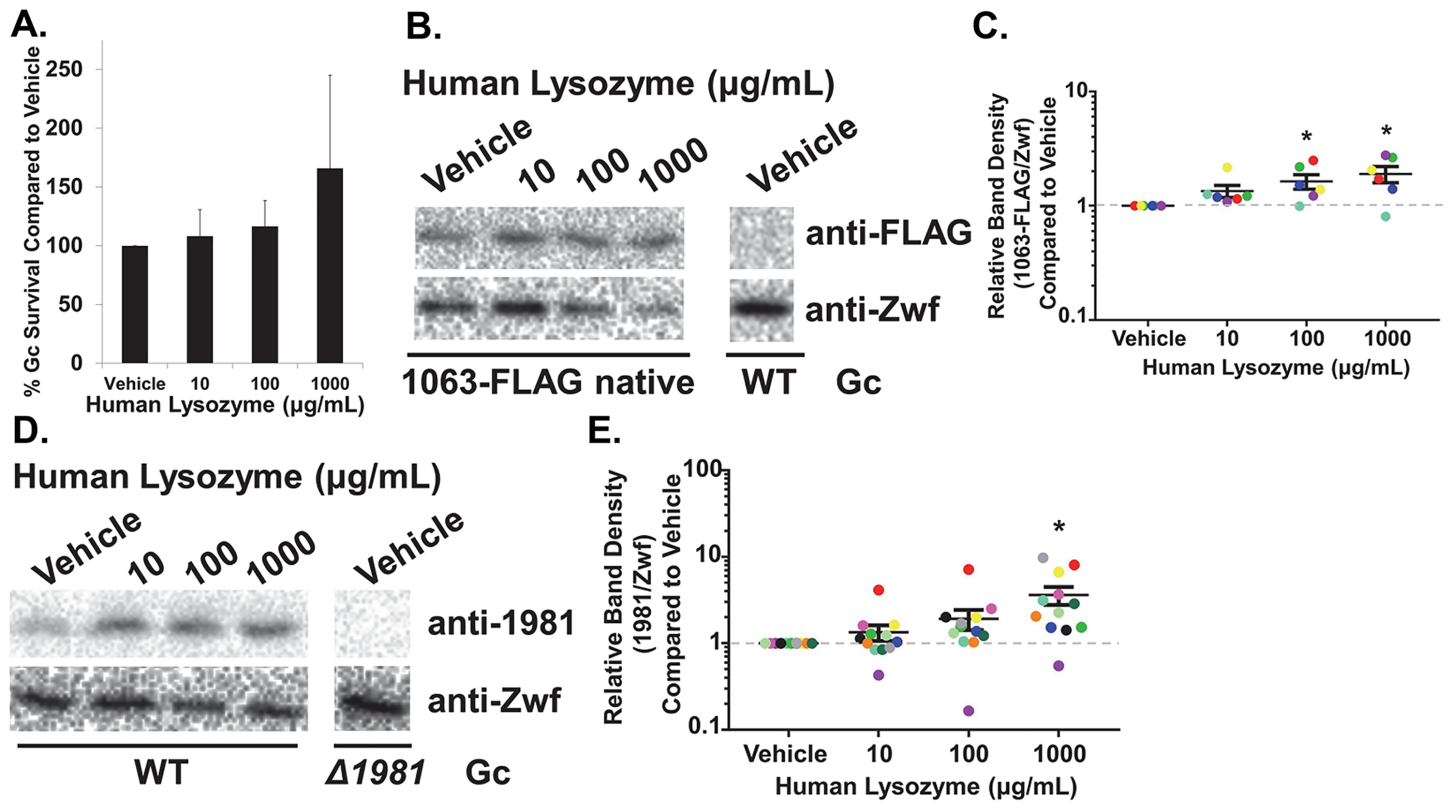
Ng_1063 and Ng_1981 protein is increased upon exposure to lysozyme. A. WT Gc was exposed to the indicated concentrations of lysozyme for 3 hr (see Materials and methods). Gc survival was determined as in Fig 2B. *n* = 3 biological replicates. Values are represented as the mean ± SEM. B. *1063*(WT)-FLAG native or WT Gc were exposed to vehicle or lysozyme at indicated concentrations for 3 hr before harvesting protein. Blots were probed with anti-FLAG antibody, and then stripped and reprobed with anti-Zwf antibody as a loading control. Shown is 1 representative immunoblot of 6 biological replicates. C. The density of Ng_1063-FLAG and Zwf bands were quantified using ImageJ. For each treatment, the ratio of Ng_1063-FLAG to Zwf density was determined and expressed relative to the matched vehicle-treated samples in that experiment (set to an arbitrary value of 1). Each biological replicate is indicated by a different colored circle. Data are shown as mean ± SEM. *p < 0.05; two tailed *t*-test, *n* = 6 biological replicates from 2 independent experiments. D. WT or *Δ1981* Gc were exposed to lysozyme and processed as in Fig. 4B. Blots were probed with anti-r1981 antisera, and then stripped and reprobed with anti-Zwf antibody as a loading control. Shown is 1 representative immunoblot of 12 biological replicates. E. Relative densities of Ng_1981 and Zwf protein were determined and presented as in Fig. 4C. *p < 0.05; two tailed *t*-test, *n* = 12 biological replicates from 4 independent experiments.

In some bacteria, exposure to lysozyme induces a signaling cascade that results in upregulation of lysozyme resistance factors [38–41]. Therefore, we next examined whether treating Gc with lysozyme under sublethal conditions (see Materials and methods and Fig 4A) affected Ng_1063 and Ng_1981 protein abundance. To do so, we engineered a Gc strain carrying *1063*(WT)-FLAG at its native locus to probe for Ng_1063 using the anti-FLAG antibody. There was a significant increase in Ng_1063 protein from Gc treated with lysozyme for 3 hrs, compared with vehicle (Fig 4B and 4C). Similarly, using anti-r1981 antisera, Ng_1981 protein was significantly increased upon lysozyme treatment, compared to vehicle (Fig 4D and 4E). On average, Ng_1063 and Ng_1981 protein were increased 2-fold and 3.5-fold, respectively, in Gc treated with 1 mg/mL lysozyme (Fig 4C and 4E).

Taken together, these findings indicate that both Ng_1063 and Ng_1981 interact with lysozyme in intact Gc, and Gc alters expression of Ng_1063 and Ng_1981 proteins after exposure to lysozyme.

### Ng_1063 and Ng_1981 are critical for fully protecting Gc from killing by lysozyme

**Fig 5:**
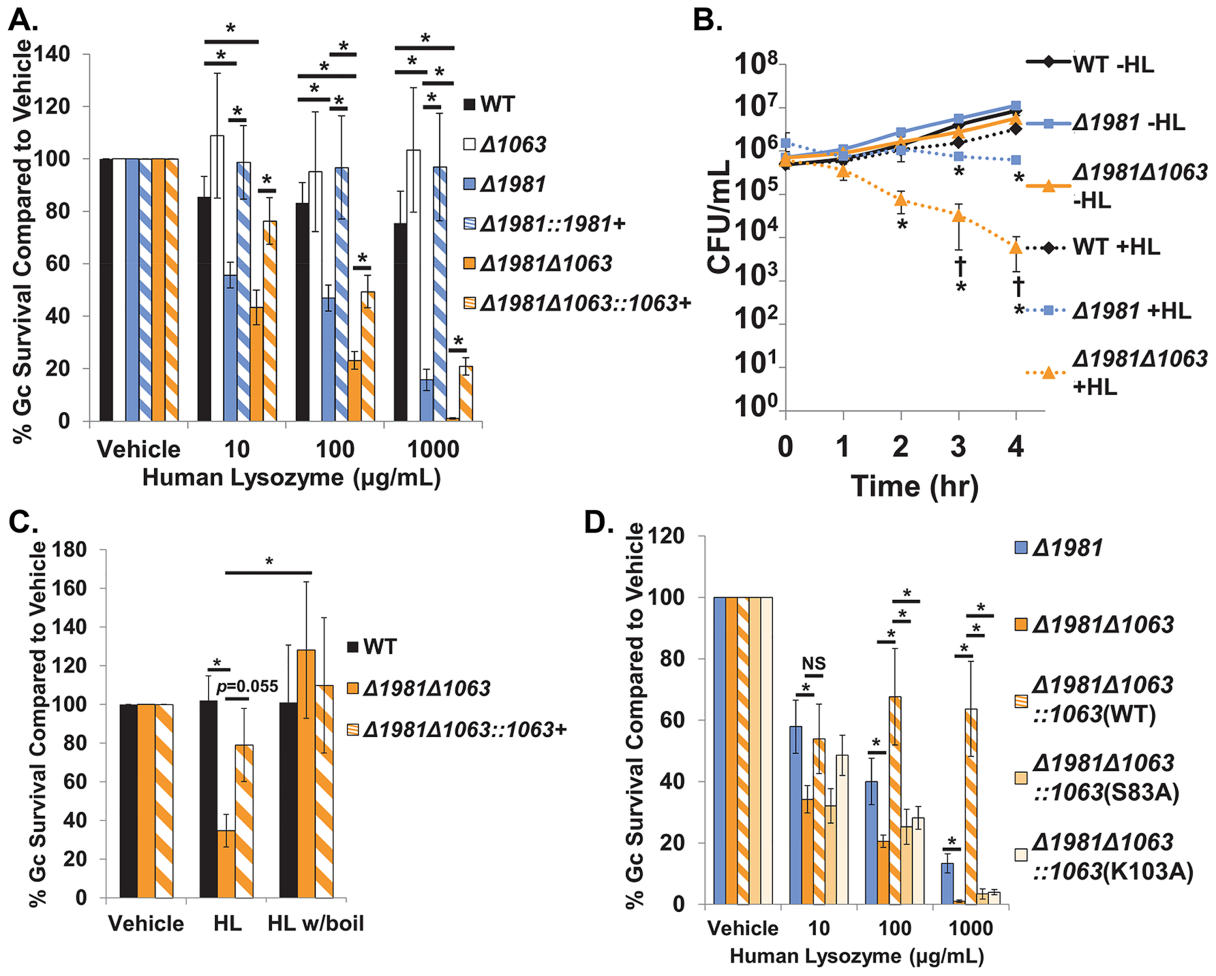
Ng_1063 and Ng_1981 are important for Gc survival from human lysozyme. A. WT, *Δ1981*, *Δ1981*::*1981*+ complement, *Δ1981Δ1063*, and *Δ1981Δ1063*::*1063*+ complement Gc were exposed to human lysozyme for 3 hr. Gc survival was determined as in Fig 2B. *p < 0.05; two tailed *t*-test, *n* = 3–31 biological replicates. B. WT, *Δ1981*, and *Δ1981Δ1063* Gc were exposed to 1,000 μg/mL human lysozyme (HL), and CFU/mL was determined over time. CFU were enumerated at the indicated times. *p < 0.05 comparing *Δ1981* or *Δ1981Δ1063* to WT, and **†***p* < 0.05 comparing *Δ1981Δ1063* to *Δ1981*; two tailed *t*-test, *n* = 3–6 biological replicates. C. WT, *Δ1981Δ1063*, and *Δ1981Δ1063*::*1063*+ complement Gc were exposed for 3 hr to 10 μg/mL human lysozyme (HL) with or without boiling for 1 hr to eliminate enzymatic activity. Gc survival was determined as in Fig 2B. *p < 0.05; two tailed *t*-test, *n* = 5–9 biological replicates. D. *Δ1981*, *Δ1981Δ1063*, *Δ1981Δ1063*::*1063*(WT)-FLAG complement, *Δ1981Δ1063*::*1063*(S83A)-FLAG complement, and *Δ1981Δ1063*::*1063*(K103A)-FLAG complement Gc were exposed to the indicated concentrations of human lysozyme for 3 hr. Gc survival was determined as in Fig 2B. NS, not significant. *p < 0.05; two tailed *t*-test, *n* = 7–10 biological replicates. All values are represented as the mean ± SEM.

We next took a genetic approach to define how Ng_1063 and Ng_1981, singly and in combination, affect Gc resistance to killing by lysozyme. To our surprise, survival of *Δ1063* mutant Gc was equivalent to WT after exposure to lysozyme (Fig 5A). One possible explanation for this phenotype is that the outer membrane barrier of Gc limits the access of lysozyme to the periplasm where Ng_1063 could be functioning; however, loss of *ng1063* had no effect on bacterial survival compared with parent Gc under the following envelope-compromising conditions: subinhibitory concentrations of the pore-forming cationic antimicrobial peptide LL-37 (S2A and S2B Fig); membrane-destabilizing treatment with EDTA (S2C Fig); and genetically-induced loss of envelope integrity using the *ΔltgAΔltgD* mutant (S3 Fig). In contrast, at the same concentrations of lysozyme used for the *Δ1063* mutant, *Δ1981* mutant Gc was significantly compromised for survival compared to WT bacteria, reaching a near 5-fold decrease in survival when exposed to 1 mg/mL lysozyme (Fig 5A). Complementation with *ng1981* restored *Δ1981* mutant survival to WT levels (Fig 5A). Loss of *ng1981* also reduced survival of the *ΔltgAΔltgD* mutant after exposure to lysozyme, an observation that highlights how multiple, mechanistically distinct factors contribute to lysozyme resistance in Gc (S3 Fig). Thus, Ng_1981 is confirmed as an important factor for Gc defense against lysozyme.

Importantly, deletion of both *ng1063* and *ng1981* markedly reduced Gc survival in the presence of lysozyme in a concentration-dependent and time-dependent manner (Fig 5A and 5B). The sensitivity to lysozyme in *Δ1981Δ1063* mutant Gc was greater than either single mutant alone, and this effect was particularly apparent at higher concentrations of lysozyme (Fig 5A and 5B). As seen in Fig 5B, recoverable CFU from *Δ1981Δ1063* mutant Gc was reduced 100-fold compared to *Δ1981* single mutant Gc after 4 hrs exposure to lysozyme. Similarly, loss of both *ng1063* and *ng1981* significantly impaired the survival of *ΔltgAΔltgD* mutant Gc after lysozyme exposure, in comparison to loss of *ng1981* alone (S3 Fig). Complementation with *ng1063* in *Δ1981Δ1063* mutant Gc restored survival to the level of the single *Δ1981* mutant (Fig 5A). In the absence of lysozyme, single and double mutants grew similarly to WT Gc (Fig 5B). These findings indicate that in addition to Ng_1981, Ng_1063 is also important for Gc defense against lysozyme. Moreover, these findings shed light on the biological activities of Ng_1063 and Ng_1981 in Gc, where Ng_1981 can fully compensate for loss of *ng1063*, yet Ng_1063 cannot fully compensate for loss of *ng1981*.

Lysozyme can kill bacteria through its hydrolase activity and through an enzyme-independent mechanism that relies on its cationicity [16, 17]. We investigated if Ng_1063 and Ng_1981, like other proteinaceous inhibitors of lysozyme, defend Gc from the enzymatic activity of lysozyme. To this end, we exposed *Δ1981Δ1063* mutant Gc to lysozyme that had been boiled, eliminating its enzymatic activity [22, 42]. Survival of the *Δ1981Δ1063* mutant was unaltered by boiled lysozyme compared to WT Gc (Fig 5C), confirming that Ng_1063 and Ng_1981 inhibit the enzymatic activity of lysozyme.

We next tested the ability of Ng_1063 and Ng_1981 to defend Gc against other peptidoglycan-degrading enzymes (i.e., peptidoglycan muramidase activity) (S4A Fig). *Δ1981Δ1063* mutant Gc was significantly reduced over 6-fold in survival after incubation with the chicken egg white c-type lysozyme, compared to WT, and survival was rescued by overexpression of *ng1063* (S4B Fig). At the concentrations tested, the single *Δ1981* mutant was unaffected by chicken egg white lysozyme (S4B Fig). In contrast, in the presence of mutanolysin, a distinct bacteria-derived muramidase (S4A Fig), survival of *Δ1981* and *Δ1981Δ1063* mutant Gc was equivalent to WT (S4C Fig). Mutanolysin was active in this setting, as evidenced by the reduced survival of *ΔltgAΔltgD* mutant Gc (S4C Fig) [22]. These findings indicate that Ng_1063 and Ng_1981 can inhibit some but not all muramidases, possibly owing to co-evolution of Gc with humans, which possess one c-type lysozyme.

Residues S89 and K103 of *P*. *aeruginosa* MliC interact with the active site of lysozyme and are required for lysozyme inhibition (see Fig 1) [36]. Thus, we evaluated if the corresponding residues in Ng_1063, S83 and K103, similarly contributed to lysozyme inhibition by complementing *Δ1981Δ1063* Gc with C-terminal 3X-FLAG-tagged Ng_1063, in which each of these residues was replaced with an alanine. Immunoblotting with a FLAG antibody showed equivalent expression between Ng_1063 WT, S83A, and K103A variants in the *Δ1981Δ1063* mutant upon IPTG induction (S5 Fig). In the presence of lysozyme, *Δ1981Δ1063* mutant Gc expressing WT Ng_1063-FLAG exhibited a significant, greater than 60-fold increase in survival, which was not reproduced with expression of either S83A or K103A Ng_1063-FLAG (Fig 5D). This finding suggests that these residues are important for the lysozyme inhibitory activity of Ng_1063.

A MUSCLE alignment between Ng_1063 and Ng_1981 shows possible conservation of the MliC inhibitory serine (S76 in Ng_1981) and lysine residues (K99 in Ng_1981) (S1A Fig). We previously found that the *N*. *meningitidis* homolog to Ng_1981, NMB_2095, exhibits structural homology to MliC/PliC inhibitors, yet computational docking models between NMB_2095 and lysozyme predicts a mode of binding that differs from MliC/PliC inhibitors [25]. Because mutation of residues in NMB_2095 that were predicted to interact with lysozyme (i.e., Asn79, Tyr84, and Gly95) failed to alter its inhibitory activity [25], we investigated the role of S76 and K99 in Ng_1981 inhibition of lysozyme. We complemented *Δ1981* mutant Gc with C-terminal 3X-FLAG-tagged Ng_1981, where S76 and K99 were replaced with an alanine residue. We observed equivalent expression of Ng_1981(WT)-FLAG, Ng_1981(S76A)-FLAG, and Ng_1981(K99A)-FLAG by immunoblot upon IPTG induction (S1D Fig). Complementation with either WT, S76A, or K99A versions of *ng1981* was sufficient to significantly increase *Δ1981* mutant survival in the presence of lysozyme (S1E Fig). This finding supports our previous conclusions that Ng_1981 and its homolog NMB_2095 behave differently from MliC/PliC inhibitors and are thus novel inhibitors of lysozyme.

### Ng_1063 and Ng_1981 contribute to Gc defense against human sources of lysozyme that are relevant in acute gonorrhea

**Fig 6:**
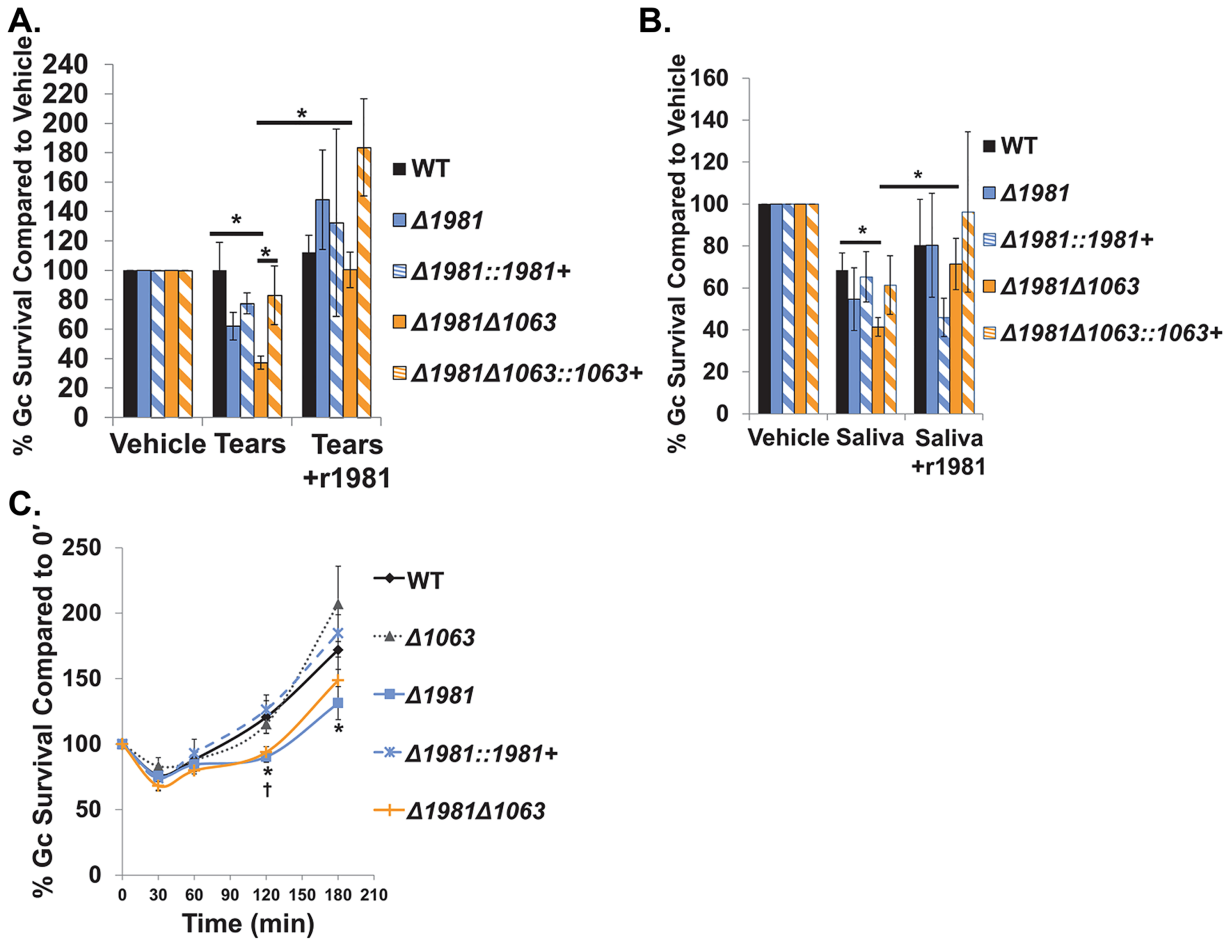
Ng_1063 and Ng_1981 are important for Gc survival from physiologically relevant sources of human lysozyme. A. WT, *Δ1981*, *Δ1981*::*1981*+ complement, *Δ1981Δ1063*, and *Δ1981Δ1063*::*1063*+ complement Gc were exposed to pooled human tears (diluted to 0.01X) that had been pretreated with recombinant 1981 (r1981) or mock-treated for 3 hr. Gc survival was determined as in Fig 2B. *p < 0.05; two tailed *t*-test *n* = 4–9 biological replicates. B. WT, *Δ1981*, *Δ1981*::*1981*+ complement, *Δ1981Δ1063*, and *Δ1981Δ1063*::*1063*+ complement Gc were exposed to pooled human saliva (diluted to 0.05) that had been pretreated with r1981 or mock-treated for 3 hr. Gc survival was determined as in Fig 2B. *p ≤ 0.05; two tailed *t*-test, *n* = 4–9 biological replicates. C. Adherent IL-8-treated primary human neutrophils were exposed to WT, *Δ1063*, *Δ1981*, *Δ1981*::*1981*+ complement, and *Δ1981Δ1063* Gc at an MOI of 1. At indicated time points, CFU were enumerated from neutrophil lysates. Percent Gc survival was determined by dividing CFU/mL at each time point by CFU/mL at 0 min. *p ≤0.05 for *Δ1981* compared to WT or *Δ1981*::*1981+* complement and †*p* ≤0.05 for *Δ1981Δ1063* compared to WT; two tailed *t*-test, *n* = 3–17 independent experiments. All values are represented as the mean ± SEM.

Mucosal sites that are colonized by Gc are bathed in fluids with high concentrations of lysozyme, including tears (2 mg/mL) and saliva (0.12 mg/mL) [13]. We therefore tested the possibility that Ng_1063 and Ng_1981 contribute to Gc survival when exposed to pooled human tears or pooled human saliva. Compared to WT Gc, *Δ1981Δ1063* mutant Gc was significantly reduced in survival in the presence of tears as well as saliva (Fig 6A and 6B). Survival of the single *Δ1981* mutant was equivalent to WT at the dilutions tested (Fig 6A and 6B). We confirmed the secretions contained active lysozyme by their ability to lyse *M*. *luteus* (S6A and S6B Fig). Pretreatment of the secretions with r1981 was sufficient to inhibit the lytic activity of secretions against *M*. *luteus* (S6A and S6B Fig), and pretreatment with r1981 restored *Δ1981Δ1063* mutant survival to WT levels (Fig 6A and 6B).

Neutrophils are another abundant source of lysozyme and are heavily recruited to sites of Gc infection [14, 15]. Thus, we tested if Ng_1063 and Ng_1981 are important for Gc survival from adherent, interleukin 8-treated primary human neutrophils, a model to recapitulate the physiological state of tissue-migrated neutrophils during gonorrheal disease [43]. *Δ1981* mutant Gc exhibited a modest but statistically significant reduction in survival in the presence of human neutrophils, compared to WT and *ng1981* complemented Gc (Fig 6C). In contrast, survival of *Δ1063* Gc was equivalent to WT after exposure to neutrophils, and *Δ1981Δ1063* mutant Gc was equally sensitive to neutrophils as the single *Δ1981* mutant (Fig 6C). Under the conditions tested, Gc resides in a phagosome that exhibits limited fusion with neutrophil primary granules, which contain lysozyme [14, 15, 44, 45]. In comparison with *Δ1981Δ1063* mutant Gc, *ΔltgAΔltgD* mutant Gc is markedly sensitive to even small amounts of lysozyme. Because we previously linked lysozyme sensitivity of the *ΔltgAΔltgD* mutant with increased killing by human neutrophils [22], we next tested whether overexpression of *ng1063* or *ng1981* enhanced *ΔltgAΔltgD* survival in the presence of human neutrophils, as was the case *in vitro* with purified human lysozyme (see Fig 2C and S1C Fig). However, neither overexpression of *ng1063* nor *ng1981* was sufficient to rescue the survival defect of *ΔltgAΔltgD* mutant Gc in the presence of human neutrophils, pointing to factors in addition to lysozyme for the sensitivity of *ΔltgAΔltgD* mutant to neutrophils (S7 Fig).

Together, these findings suggest that Ng_1063 and Ng_1981 help defend Gc from physiologically relevant sources of lysozyme that would be encountered in its obligate human host.

### Differential localization of Ng_1063 and Ng_1981 in Gc

**Fig 7:**
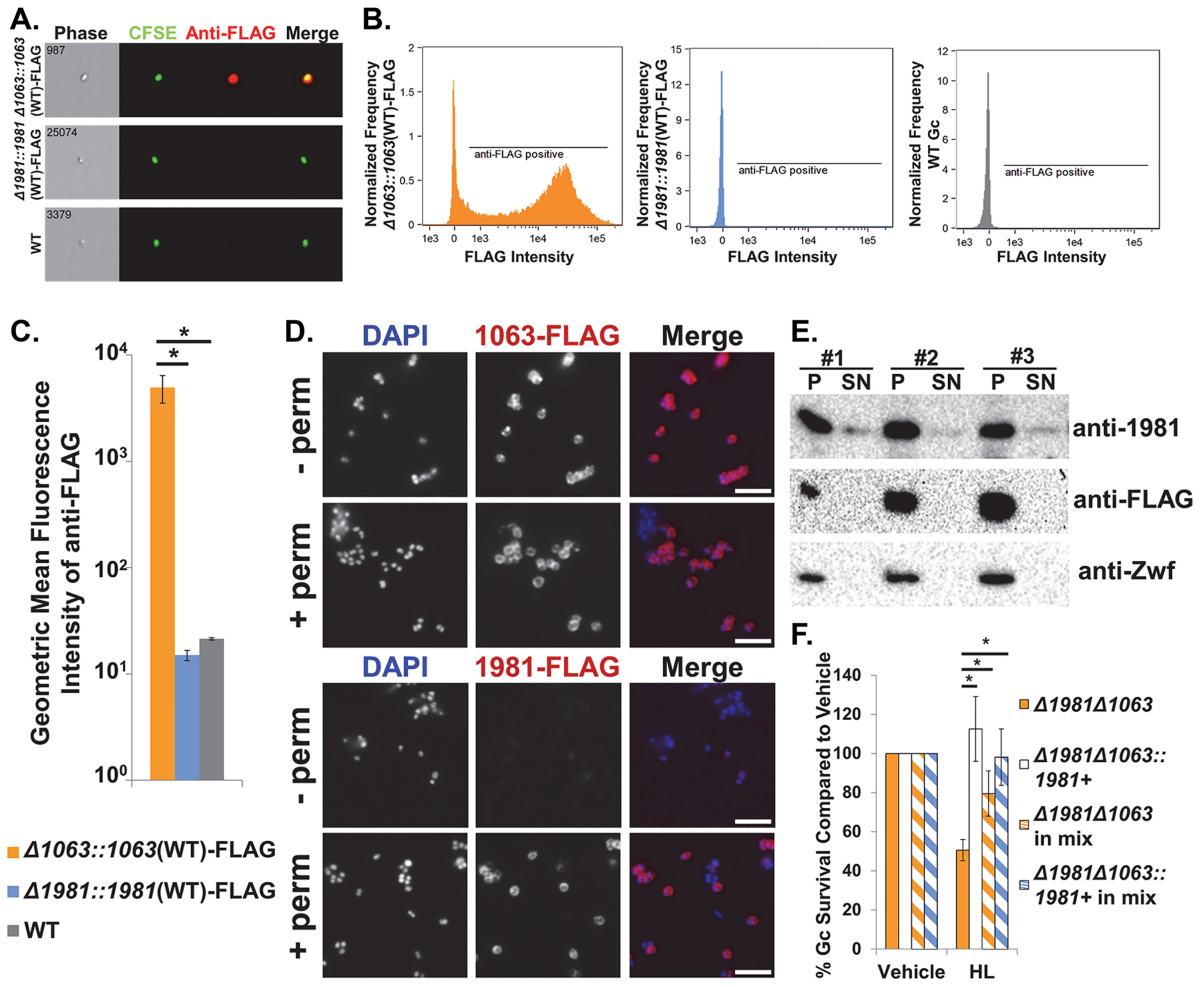
Ng_1063 and Ng_1981 localization in Gc. A. Representative images of 1063-FLAG and 1981-FLAG surface expression via imaging flow cytometry. Live *Δ1063*::*1063*(WT)-FLAG and *Δ1981*::*1981*(WT)-FLAG Gc were labelled with CFSE and subsequently stained with mouse anti-FLAG, followed by goat anti-mouse AlexaFluor647. WT Gc expressing no FLAG-tagged proteins was used as a negative control. *n* = 4 biological replicates. B. Histogram of anti-FLAG/AlexaFluor647 fluorescence intensity for *Δ1063*::*1063*(WT)-FLAG, *Δ1981*::*1981*(WT)-FLAG, and WT Gc from Fig. 7A. Shown is 1 representative of 4 biological replicates. C. Geometric mean fluorescence intensity of anti-FLAG-AlexaFluor647 for *Δ1063*::*1063*(WT)-FLAG, *Δ1981*::*1981*(WT)-FLAG, and WT Gc from Fig. 7A. Values are represented as the mean ± SEM from 4 biological replicates. *p < 0.05; two tailed *t*-test. D. Immunofluorescence micrographs of 1063-FLAG or 1981-FLAG in Gc with or without cell permeabilization. After paraformaldehyde fixation, *Δ1063*::*1063*(WT)-FLAG and *Δ1981*::*1981*(WT)-FLAG Gc were permeabilized with methanol and Triton-X-100 (+ perm) or left untreated (- perm). Bacterial DNA was stained with DAPI (blue) and FLAG proteins were detected with an anti-FLAG antibody (red). Scale bar, 5μm. Micrographs are from 1 representative of 3 independent experiments. E. Gc expressing *1063*(WT)-FLAG under its native promoter were grown for 3 hr, at which point bacterial whole cell lysates (pellet, P) and conditioned supernatants (SN) were collected for SDS-PAGE and immunoblotting. To detect native Ng_1981 and Ng_1063 protein, blots were probed with anti-r1981 and anti-FLAG, respectively. The cytoplasmic protein Zwf (using anti-Zwf antibody) served as a loading control for proteins present in the whole cell lysate. Shown are the results from 3 biological replicates, labelled #1–3. F. *Δ1981Δ1063* mutant Gc, *Δ1981Δ1063*::*1981*+ complement Gc, and a 1:1 mix of *Δ1981Δ1063* with *Δ1981Δ1063*::*1981*+ Gc were grown for 1 hr prior to exposure to 10 μg/mL human lysozyme (HL) for 3 hr. Gc survival was calculated as in Fig 2B. *p < 0.05; two tailed *t*-test, *n* = 8 biological replicates.

Although Ng_1063 and Ng_1981 both interact with lysozyme and display a similar ability to inhibit the enzymatic activity of lysozyme, their biological activities in Gc are distinct. To gain insight into the mechanism underlying these differences, we examined the localization of Ng_1063 and Ng_1981 in Gc. Ng_1063 is a putative lipoprotein predicted to be extracellularly exposed on the surface of Gc [46], whereas Ng_1981 is predicted to be a soluble, periplasmic protein, according to LipoP 1.0 and CELLO bioinformatic analyses. To test these predictions, we assessed the surface exposure of each protein, using *Δ1063* and *Δ1981* mutant Gc complemented with IPTG-inducible WT copies of C-terminal FLAG-tagged Ng_1063 and Ng_1981, respectively. FLAG-complemented Gc were incubated with anti-FLAG antibody, and fluorescence intensity per individual bacterium was quantified by imaging flow cytometry. We detected strong surface labeling of FLAG protein from *ng1063*-FLAG complemented Gc (Fig 7A–7C). In contrast, *ng1981*-FLAG complemented Gc displayed negligible surface expression of FLAG protein, which was no different from the negative control (Fig 7A–7C).

As a complementary approach, we visualized FLAG-tagged protein expression using immunofluorescence microscopy. We observed staining with the FLAG antibody in *ng1063*-FLAG complemented Gc, but not in *ng1981*-FLAG complemented Gc under non-permeabilizing conditions, in agreement with the imaging flow cytometry data (Fig 7D). Surface expression of Ng_1981-FLAG was also not detectable using polyclonal anti-r1981 antisera (S8 Fig). When Gc was permeabilized with methanol and Triton-X-100 prior to incubation with the FLAG antibody, both *ng1063*-FLAG and *ng1981*-FLAG complemented Gc exhibited peripheral staining, suggestive of localization to the bacterial envelope (Fig 7D). Together, these findings indicate that Ng_1063, but not Ng_1981, is exposed extracellularly on the surface of Gc.

The lack of Ng_1981 surface staining was unexpected because its homolog in *N*. *meningitidis*, NMB_2095, was found to be surface-exposed, where it contributes to bacterial adhesion to epithelial cells [47]. A clue to resolving this discrepancy came from the results in Fig 3, where we observed less FLAG-tagged Ng_1981 protein than FLAG-tagged Ng_1063 protein in lysates prepared from pelleted bacteria, despite using the same overexpression system. We thus considered the possibility that a fraction of Ng_1981 is released from Gc. To directly test this possibility, we evaluated the presence of Ng_1981 and Ng_1063 protein in bacterial whole cell lysates and supernatants. We used Gc carrying *1063*(WT)-FLAG at its native locus to simultaneously detect Ng_1063-FLAG using the anti-FLAG antibody and Ng_1981 using anti-r1981 antisera. Conditioned supernatants from this strain contained Ng_1981, but not Ng_1063, while both proteins were present in the whole cell lysates (Fig 7E). In addition, we did not detect the cytoplasmic protein ZWF in conditioned supernatants (Fig 7E), providing further evidence that Ng_1981 is not released as the result of autolysis.

To test whether Ng_1981 in Gc supernatants contains functional inhibitory activity against lysozyme, we co-cultured *Δ1981Δ1063* mutant Gc with Ng_1981-complemented Gc in the *Δ1981Δ1063* background. Under these conditions, the presence of the *ng1981* complement significantly increased the survival of the non-complemented *Δ1981Δ1063* mutant after exposure to lysozyme (Fig 7F). This observation suggests that extracellularly released Ng_1981 can protect Gc from lysozyme, in keeping with our initial findings with ectopically added recombinant protein (S1 Fig).

Taken together, these results indicate that Gc produces two distinct inhibitors of lysozyme: Ng_1981 is both released extracellularly and is in the bacterial envelope, whereas Ng_1063 is OM-anchored and surface-exposed.

## Discussion

The success of Gc as a human pathogen requires that it defends itself against antimicrobial components found at mucosal surfaces. In this work, we identified Ng_1063 as a new functional homolog of the MliC-type lysozyme inhibitors in Gc. We then interrogated its functions alongside another, recently characterized lysozyme inhibitor, Ng_1981 [25]. We found that Ng_1063 and Ng_1981 interact with lysozyme in the physiological context of the bacterium, and both proteins’ expression was increased upon exposure to lysozyme. Gc lacking both *ng1063* and *ng1981* was markedly reduced in survival after exposure to human lysozyme and lysozyme-containing human secretions, and this effect was greater than either single mutant alone. Ng_1063 was exposed on the surface of Gc, while Ng_1981 was not; however, a fraction of Ng_1981 protein was released into the extracellular milieu. Based on these results, we conclude that Gc produces two distinct inhibitors of lysozyme that together confer full resistance to this abundant antimicrobial defense protein.

MliC-type inhibitors of lysozyme are periplasmic-facing lipoproteins that form an eight-stranded antiparallel β-barrel structure [31]. The structure of *P*. *aeruginosa* MliC in complex with lysozyme revealed the protrusion of two loops from the inhibitor into the active site of lysozyme, each loop contributing one key, conserved inhibitory residue (S89 and K103) [31, 36]. Our *in silico* analyses predicted that Ng_1063 is a lipoprotein with sequence and structural similarity to MliC-type inhibitors. We obtained biological support for this prediction by showing that the corresponding inhibitory residues in Ng_1063, S83 and K103, were required for its inhibitory activity. In contrast, S83 and K103 were dispensable for the interaction of Ng_1063 with lysozyme *in vivo* (Fig 3B). This finding implicates a second binding site in Ng_1063 for lysozyme, which has been shown for a shallow binding pocket consisting of Y92 and T98 in *P*. *aeruginosa* MliC [36]. Intriguingly, we found that C-terminal FLAG-tagged Ng_1063 is on the surface of Gc. Since Ng_1063 has no predicted membrane-spanning regions, the entire Ng_1063 protein is likely exposed extracellularly, where it could bind and inhibit lysozyme. This observation contrasts with most MliC-type inhibitors, which are predicted to reside within the inner leaflet of the OM, facing the periplasm [31]. Since most studies have not experimentally verified localization of MliC proteins, we anticipate other microbes produce MliC-type proteins that are also surface-exposed. It remains unclear how Ng_1063 becomes surface exposed, but a likely candidate involves the recently identified surface lipoprotein assembly modulator (Slam) [48].

Several bacterial species, including *E*. *coli*, *P*. *aeruginosa*, *Yersinia pestis*, and *Edwardsiella tarda*, produce more than one inhibitor of lysozyme, and often these proteins are non-redundant [26–28, 32–35]. Here, we found that Gc produces two proteins, Ng_1063 and Ng_1981, which both bind to and inhibit lysozyme. Each protein’s expression is increased upon exposure to lysozyme. While other bacteria regulate lysozyme resistance factors via alternative sigma factors or two component systems [38–41, 49], such regulatory networks are limited in Gc; thus how this regulation is occurring in Gc is not yet clear. Despite their shared ability to inhibit lysozyme, Ng_1063 and Ng_1981 have several distinguishable properties. First, even though Ng_1063 and Ng_1981 share overall structural similarity with MliC/PliC-type inhibitors [25], these proteins share little sequence similarity (19% identity; 35% similarity). In fact, Ng_1981 lacks conserved MliC/PliC sequence motifs [25, 31]. Further, we found that mutation of the Ng_1981 residues S76 and K99, which align with the key inhibitory residues of Ng_1063, failed to alter Ng_1981 inhibitory activity against lysozyme. Ng_1981 protein is also predicted to be soluble whereas Ng_1063 is a predicted lipoprotein. In contrast to Ng_1063 that is surface-exposed, we found evidence that Ng_1981 is present both in the Gc envelope and in the extracellular milieu. It remains unclear whether extracellular Ng_1981 is present as a soluble protein or resides within or associates with outer membrane vesicles; however, we found evidence that *ng1981* expressing Gc could rescue survival of lysozyme-susceptible Gc *in trans*, suggesting that extracellular Ng_1981 retains inhibitory activity. Future studies will investigate how Ng_1981 is released by Gc.

Gc employs both Ng_1063 and Ng_1981 for optimal defense against lysozyme, but in a non-redundant manner. Loss of *ng1981* significantly reduced Gc survival when exposed to lysozyme or human neutrophils. This finding extends upon our previous work, which showed sensitivity of the *Δ1981* mutant in a different genetic background (strain FA1090) to lysozyme [25], and suggests that the presence of Ng_1063 is insufficient to fully compensate for loss of *ng1981*. In contrast, survival of Gc lacking *ng1063* was equivalent to WT after exposure to lysozyme. Even though OM-permeabilization has been used to reveal a protective role for lysozyme inhibitors in other bacteria [34, 35], survival of *Δ1063* mutant Gc remained unaltered from WT when the Gc envelope was compromised. Although our reductionist conditions did not reveal a phenotype for the single *Δ1063* mutant, it is possible that this protein contributes to Gc survival *in vivo*, warranting future investigation. This finding implies that Ng_1981 sufficiently compensates for loss of *ng1063* under our *in vitro* conditions. The differential ability of Ng_1981 and Ng_1063 to protect Gc from lysozyme may be explained by their differential localization, as well as differences in their expression or affinity for lysozyme, all of which are subjects for future investigation. Nevertheless, the role of Ng_1063 as a barrier to lysozyme in Gc was revealed in the absence of *ng1981*, where *Δ1981Δ1063* mutant Gc exhibited increased sensitivity to lysozyme over either single mutant. Importantly, *ng1063* and *ng1981* are expressed during human infection in female patients, implying that both proteins contribute to colonization and/or pathogenesis *in vivo* [50]. Moreover, pathogenic and non-pathogenic *Neisseria* carry homologs for both Ng_1063 (S2 Table) and Ng_1981 [25], and in the Ng_1063 homologs, the S83 and K103 equivalent residues are 100% conserved (S3 Table). Since commensal *Neisseria*, like the pathogens, colonize mucosal sites, conservation of Ng_1063 and Ng_1981 amongst *Neisseria* may be important for residence at these lysozyme-rich surfaces. Together, these findings support a role for both Ng_1063 and Ng_1981 in Gc lysozyme resistance and implicate these inhibitors as important virulence determinants in Gc.

In addition to Ng_1063 and Ng_1981, Gc *O*-acetylates its peptidoglycan to directly block lysozyme-mediated hydrolysis [22, 24]. Gc also expresses two cell wall turnover proteins, LtgA and LtgD, that maintain envelope integrity, a physical barrier to lysozyme [22]. Thus, lysozyme resistance in Gc is multifactorial. Given the diverse mechanisms employed by Gc to resist lysozyme, if and how these factors are connected at the level of gene regulation or protein-protein interactions remains an open question. However, it is noteworthy that each factor contributes unequally to lysozyme resistance. For example, *O*-acetylation is dispensable for Gc survival in the presence of lysozyme, as long as envelope integrity is also maintained [22, 24]. In contrast, both an *ΔltgAΔltgD* mutant and an *Δ1981Δ1063* mutant exhibit sensitivity to low concentrations of lysozyme, where the *ΔltgAΔltgD* mutant is the most sensitive [22]. Together, these mechanistically distinct approaches underscore that lysozyme resistance in Gc is vital to the infectivity of this pathogen. By extension, these observations open the possibility of targeting such factors to enhance bacterial susceptibility to lysozyme in the human host. For instance, chemicals that interfere with Ng_1063 and Ng_1981 could help to treat infections with strains exhibiting increased resistance to traditional antibiotics. Ng_1063 and Ng_1981 are also potential vaccine candidates, based on their extracellular localization, expression during human infection, and relative conservation among Gc strains (S2 Table) [25, 50, 51]. A vaccine against Ng_1063 and Ng_1981 is attractive because antibodies raised against them could promote Gc killing by multiple mechanisms. For instance, antibody deposition on surface Ng_1063 could promote bacterial killing via opsonophagocytosis and serum bactericidal activity, while function-neutralizing antibodies against both extracellular Ng_1063 and Ng_1981 could sensitize Gc to lysozyme in mucosal secretions and in neutrophils [51]. Thus, we propose that Ng_1063 and Ng_1981 are worthy targets for consideration in the context of revitalized global efforts to develop new antibiotics and vaccines against the urgent threat of drug-resistant gonorrhea.

## Materials and methods

### Ethics statement

Human Subjects: Venous blood was collected from adult healthy human subjects with their signed informed consent. All materials collected were in accordance with a protocol (#13909) approved by the University of Virginia Institutional Review Board for Health Sciences Research.

Animals: Healthy, specific pathogen-free rabbits were immunized with r1981 by Davids Biotechnologie GmbH (Regensburg, Germany). Davids Biotechnologie GmbH has a permit from the Veterinäramt Regensburg for housing specific-pathogen free, healthy rabbits according to §11 TierSchG (Az31.4.4/ScP1). The company is registered for immunization of animals under the Aketenzeichen: AZ 2532.44/14 at the approving authority Umweltamt Regensburg/Veterinärwesen. All immunizations were done in accordance with National Institute of Health standards for animal welfare (NIH animal welfare number A5646-01).

### Bacterial strains

All Gc used in this study are in the MS11 background and are listed in S4 Table. WT Gc is a RecA+ MS11 strain nonvariably expressing the VD300 pilin [22], and *ΔltgAΔltgD* mutant Gc was used from previous work [52].

A null allele of *ng1063* was made by introducing a stop codon in-frame into the coding region of the gene. A 5’ flank was PCR amplified using the forward primer 5’AAAAATTTACATTCCTCCGGGCGGGC3’ and the mutagenesis reverse primer 5’GCACAGGCCTACAATCTAGAAACCGATACG3’ (in-frame stop codon; XbaI site). A 3’ flank was amplified by PCR using the mutagenesis forward primer 5’TTTCTAGATTGTAGGCCTGTGCCGTG3’ (XbaI site; in-frame stop codon) and the reverse primer 5’AGCAGGTTTAAAGTTGGCATTGAGCCG3’. The 5’ and 3’ flanks were combined via overlap extension PCR, and the resulting product was introduced into Gc by spot transformation [53]. Bacteria from the transformation were screened by PCR followed by digestion with XbaI, and positive transformants were confirmed by sequencing the *ng1063* allele. The *ng1981* mutant was made by transforming Gc with pGEM-*Δ1981*, where 1981 is disrupted with a kanamycin cassette [25]. Transformants were selected on 50 μg/mL kanamycin and positive transformants confirmed by PCR.

To make the *ng1981* complementation construct, *ng1981* was PCR amplified using the forward primer 5’CCCCCGGGCGCCTTTTTACAAAC3’ (SmaI site) and reverse primer 5’GGAACCGCGGAAAAACAGCGTTTTCAG3’ (SacII site). The resulting product was digested with SmaI and SacII and ligated into the isopropyl β-D-1-thiogalactopyranoside (IPTG)-inducible complementation plasmid pKH35 [54]. The resulting plasmid (pKH35_*1981*) was used to spot transform Gc, where *ng1981* was integrated between the *lctP* and *aspC* chromosomal loci. Bacteria were selected with 8 μg/mL chloramphenicol and confirmed by DNA sequencing. To make the *ng1063* complementation construct, *ng1063* was PCR amplified using the forward primer 5’ACATAGCCGCGGGCTTTAATGTG3’ (SacII site) and the reverse primer 5’ GACAGGAGATATCCAGAACGAAACG3’ (EcoRV site). The resulting product was digested with SacII and EcoRV and ligated into the anhydrotetracycline (AT)-inducible complementation plasmid pMR68 [55]. The resulting plasmid (pMR68_*1063*) was used to spot transform Gc, where *ng1063* was integrated between the *iga* and *trpB* chromosomal loci. Transformants were selected for using 10 μg/mL erythromycin and confirmed by DNA sequencing.

To make the *ng1063*(S83A) point mutant, a 5’ flank was PCR amplified with the forward primer 5’ACATAGCCGCGGGCTTTAATGTG3’ (SacII site) and the reverse primer 5’GTTCGCCCGCTGCGGCAACGTC3’ (codon for alanine). A 3’ flank was PCR amplified with the forward primer 5’GACGTTGCCGCAGCGGGCGAAC3’ (codon for alanine) and the reverse primer 5’GACAGGAGATATCCAGAACGAAACG3’ (EcoRV site). The 5’ and 3’ flanks were combined via overlap extension PCR. The resulting product was digested with SacII and EcoRV, and ligated into pMR68 to make pMR68_*1063*(S83A). To make the *ng1063*(K103A) point mutant, a 5’ flank was PCR amplified with the forward primer 5’ACATAGCCGCGGGCTTTAATGTG3’ (SacII site) and the reverse primer 5’ CTTCGCCGCCCGCCTGGTGCCAC3’ (codon for alanine). A 3’ flank was PCR amplified using the forward primer 5’GTGGCACCAGGCGGGCGGCGAAG3’ (codon for alanine) and the reverse primer 5’ GACAGGAGATATCCAGAACGAAACG3’ (EcoRV site). The 5’ and 3’ flanks were combined via overlap extension PCR. The resulting product was digested with SacII and EcoRV, and ligated into pMR68 to make pMR68_*1063*(K103A).

To make the *ng1981*-3XFLAG construct, *ng1981* was PCR amplified using the forward primer 5’CCCCCGGGCGCCTTTTTACAAAC3’ (SmaI site) and the reverse primer 5’TTGAATTCACGTGGGGAACAGTCTTTG3’ (EcoRI site). The resulting product was digested with SmaI and EcoRI, and then ligated into pMR100, a C-terminal 3XFLAG vector [56], to make pMR100_*1981*(WT). For additional 3’ homology, the 3’ region for *ng1981* was PCR amplified using the forward primer 5’ GGCAAGCTTAAACAGCGTTTTCATTTCTG3’ (HindIII site) and the reverse primer 5’ GGCTCGAGGCCGCGGTCATTAAAAAAGAC3’ (XhoI site and SacII site). The resulting product was digested with HindIII and XhoI, and then ligated into pMR100_*1981*(WT). The resulting plasmid, pMR100_*1981*(WT)_3’homology, was digested with SmaI and SacII to release the *1981*(WT)-3XFLAG-3’homology fragment, which was subsequently ligated into pKH35 to make pKH35_*1981*(WT)_3’homology. pKH35_*1981*(WT)_3’homology was used to spot transform Gc. Transformants were selected with chloramphenicol and were confirmed by DNA sequencing.

To make the *ng1063*(WT)-3XFLAG, *ng1063*(S83A)-3XFLAG, and the *ng1063*(K103A)-3XFLAG constructs, *ng1063* was PCR amplified from MS11 genomic DNA, pMR68_*1063*(S83A) plasmid DNA, and pMR68_*1063*(K103A) plasmid, respectively, with the forward primer 5’ACATAGCCCGGGGCTTTAATGTG3’ (SmaI site) and the reverse primer 5’TTGAATTCACGGGCGCGGCAGGAAGTTTC3’ (EcoRI site). The resulting products were digested with SmaI and EcoRI and each ligated into pMR100 to make pMR100_*1063*(WT), pMR100_*1063*(S83A), and pMR100_*1063*(K103A). For additional 3’ homology, the 3’ region of *ng1063 w*as PCR amplified using the forward primer 5’AAAAAGCTTAGCCTGTTTGAACCGCCG3’ (HindIII site) and the reverse primer 5’ GACTCGAGCCCGCGGACTTTAGGC3’ (XhoI site and SacII site). The resulting product was digested with HindIII and XhoI, and then ligated into pMR100_*1063*(WT), pMR100_*1063*(S83A), and pMR100_*1063*(K103A). The resulting plasmids, pMR100_*1063*(WT)_3’homology, pMR100_*1063*(S83A)_3’homology, and pMR100_*1063*(K103A)_3’homology, were digested with SmaI and SacII to release the *1063*-3XFLAG-3’homology fragments, which were subsequently ligated into pKH35 to make pKH35_*1063*(WT)_3’homology, pKH35_*1063*(S83A)_3’homology, and pKH35_*1063*(K103A)_3’homology. Gc were spot transformed with these plasmids. Transformants were selected with chloramphenicol and were confirmed by DNA sequencing.

To make the *ng1981*(S76A)-3XFLAG construct, a 5’ flank of *ng1981* was PCR amplified from MS11 genomic DNA using the forward primer 5’CCCCCGGGCGCCTTTTTACAAAC3’ (SmaI site) and the reverse primer 5’ GTCCATATTGTCCGCTTTATCCAAATTG3’ (codon for alanine). A 3’ flank of ng1981 was PCR amplified using the forward primer 5’CAATTTGGATAAAGCGGACAATATGGAC3’ (codon for alanine) and the reverse primer 5’TTGAATTCACGTGGGGAACAGTCTTTG3’ (EcoRI site). The 5’ and 3’ flanks were combined using overlap extension PCR. The resulting amplicon was digested with SmaI and EcoRI for replacement of WT *ng1981* in the pMR100_*1981*(WT)_3’homology backbone to make pMR100_*1981*(S76A)_3’homology. pMR100_*1981*(S76A)_3’homology was subsequently digested with SmaI and SacII as above for insertion into pKH35 to make pKH35_*1981*(S76A)_3’homology. To make the *ng1981*(K99A)-3XFLAG construct, a 5’ flank of *ng1981* was PCR amplified from MS11 genomic DNA using the forward primer 5’CCCCCGGGCGCCTTTTTACAAAC3’ (SmaI site) and the reverse primer 5’GTTTGCGGTAGGACGCGCTGTCCATTG3’ (codon for alanine). A 3’ flank of ng1981 was PCR amplified using the forward primer 5’CAATGGACAGCGCGTCCTACCGCAAAC3’ (codon for alanine) and the reverse primer 5’TTGAATTCACGTGGGGAACAGTCTTTG3’ (EcoRI site). Overlap extension PCR was used to combine the 5’ and 3’ flanks, and the resulting amplicon was digested with SmaI and EcoRI to make pMR100_*1981*(K99A)_3’homology as above. pMR100_*1981*(K99A)_3’homology was digested with SmaI and SacII and inserted into pKH35 to make pKH35_*1981*(K99A)_3’homology. Gc were transformed with the pKH35 constructs via spot transformation, and transformants were selected for with chloramphenicol and were confirmed by DNA sequencing.

To replace the native *ng1063* gene with *ng1063* with a C-terminal 3X-FLAG tag, the 5’ flank of *ng1063* was PCR amplified from MS11 genomic DNA with the forward primer 5’AAAAATTTACATTCCTCCGGGCGGGC3’ and the reverse primer 5’CGGTCAGCGCGAAAAACCTGGTATT3’. The 3’ flank was PCR amplified from the pKH35_1063(WT)_3’homology plasmid with the forward primer 5’AATACCAGGTTTTTCGCGCTGACCG3’ and the reverse primer 5’TCTTGCAAGCGTTGGCAAACAGC3’. The 5’ and 3’ flanks were combined via overlap extension PCR, and the resulting product was spot transformed into Gc. Transformants were screened by PCR using the forward primer 5’AATACCAGGTTTTTCGCGCTGACCG 3’ and the reverse primer 5’AGCAGGTTTAAAGTTGGCATTGAGCCG3’, and positive transformants confirmed by DNA sequencing.

### Bacterial growth conditions

Piliated, opa-negative Gc were grown on Gonococcal Medium Base (GCB, Difco) plus Kellogg’s supplements [57] at 37°C with 5% CO_2_ (v/v). Gc was inoculated into liquid medium (GCBL with Kellogg’s supplements and NaHCO_3_) and repeatedly diluted until Gc reached mid-logarithmic stage, as described [58]. For experiments using human neutrophils, the absence of Opa expression in Gc was confirmed by Western blot with the 4B12 pan-Opa antibody. For IPTG- and AT-inducible constructs, 1 mM IPTG and 10 ng/mL AT, respectively, were added to Gc growing in liquid culture for at least 5 hr.

### *In silico* analyses

To compare protein sequences, the MUltiple Sequence Comparison by Log-Expectation (MUSCLE, http://www.ebi.ac.uk/Tools/msa/muscle/) tool was used. For alignment of *ng1063* alleles, the Clustal omega tool was used. To determine percent identity and percent similarity between proteins, the Sequence Manipulation Suite (Ident and Sim; http://www.bioinformatics.org/sms2/ident_sim.html) feature was used on the MUSCLE alignment between two proteins in question, with signal sequences included. Protein signal sequences were predicted using the LipoP 1.0 Server (http://www.cbs.dtu.dk/services/LipoP/), and envelope localization was predicted using CELLO (subCELlular LOcalization predictor; http://cello.life.nctu.edu.tw/). The protein sequence of Ng_1063 (MS11), excluding the predicted signal sequence, was used for structure prediction via the PHYRE^2^ server (www.sbg.bio.ic.ac.uk/phyre2). The predicted Ng_1063 structure was aligned with the known structure of MliC from *P*. *aeruginosa* in complex with hen egg white lysozyme (PDB 3f6z, [36]) using PyMOL Molecular Graphics System. For allelic sequence comparisons across *Neisseria*, the NEIS1425 (*ng1063*) allele was analyzed on December 1, 2017 using the PubMLST database (http://pubmlst.org/perl/bigsdb/bigsdb.pl?db=pubmlst_neisseria_isolates).

### Cloning, expression, and purification of recombinant protein

The *ng1063* gene sequence, optimized for *E*. *coli* expression and encoding the entire coding sequence for *ng1063* (NEIS1425, http://pubmlst.org/neisseria/, 381 bp), was synthesized *in vitro* (GeneArt, Invitrogen). The *ng1063* gene was cloned into the pET22b(+) system (Novagen) and inserted between the NdeI and XhoI restriction sites fused to a C-terminal hexa-histidine tag. The resulting recombinant plasmid, pET22b::*1063*(WT), was transformed into *E*.*coli* DH5α cells for plasmid amplification, and subsequently transformed into competent *E*. *coli* BL21 (DE3) pLysS (NEB) cells for protein expression. The transformants were cultured in LB broth at 37°C to mid-logarithmic phase, and expression of recombinant protein was induced by addition of IPTG to a final concentration of 1 mM. After growth at 37°C for an additional 4 h, the cells were harvested and insoluble recombinant Ng_1063 (r1063) protein was purified by nickel iminodiacetic acid (Ni-IDA) affinity chromatography under denaturing conditions. Bound protein was eluted using 100 mM NaH_2_PO_4_, 10 mM Tris-HCl, 6 M GuHCl and 250 mM imidazole buffer, pH 8.0, precipitated with 5% v/v Trichloroacetic acid (TCA), and subsequently resuspended in phosphate buffered saline (PBS), with 0.5% w/v SDS for solubilization. Protein concentration was determined using the BCA^™^ Protein Assay (Pierce). The molecular mass of mature r1063-His-tag without the leader peptide sequence (predicted by SignalP 4.1 Server) is 12.5 kDa, as confirmed by SDS-PAGE. Recombinant MIP (rMIP) and recombinant Ng_1981 (r1981) were prepared as described in [25, 59].

### Generation of rabbit antisera to r1981

Rabbits (*n* = 2) were hyper-immunized subcutaneously with r1981 using the services of David Biotechnologie GmbH, Regensburg, Germany. Rabbits were immunized with r1981 (100 μg per dose per rabbit) emulsified in Freund’s Complete Adjuvant for the primary injection (day 0) and Freund’s Incomplete Adjuvant for a subsequent four injections at ~14 day intervals, with terminal bleeding on day 63. All sera were stored at -20°C until needed.

### *Micrococcus luteus* lysis assays with lysozyme

Lysis kinetics of freeze-dried *Micrococcus luteus* cells (ATCC 4698) were performed as previously described [25]. *M*. *luteus* cells were exposed to 2 μg/ml human lysozyme (Sigma) in the absence or presence of increasing concentrations of PBS-diluted r1063. Bacterial lysis was measured by the change in optical density (OD_595_) at 25 °C over time, using a spectrophotometer (microplate reader). Pooled human tears (filter sterilized, LEE Biosolutions) were diluted to 0.1X in H_2_O and pretreated with 200 μg/mL recombinant 1981 (r1981) [25], or vehicle (H_2_O), for 20 min at 37°C. Pooled human saliva (Lee Biosolutions) was similarly diluted to 0.5X with 125 μg/mL r1981, or vehicle (H_2_O), for 20 min at 37°C. *M*. *luteus*, which had been prepared as previously described [22], was exposed to pretreated secretions for a final concentration of 0.01X tears (20 μg/mL r1981) and 0.05X saliva (12.5 μg/mL r1981). Bacterial lysis was measured over time at OD_450_ at 37°C using a Victor3 multilabel plate reader (Perkin-Elmer).

### Antimicrobial protein susceptibility

Mid-logarithmic phase Gc was suspended in 0.5X GCBL (diluted with H_2_O; Kellogg’s supplements and NaHCO_3_ were also at 0.5X), with IPTG and AT, if necessary, prior to exposure to the antimicrobial protein at 37°C with 5% CO_2_ (v/v). The final concentration of Gc with lysozyme was 5x10^5^ CFU/mL, unless otherwise indicated. Gc survival is expressed as the percent of Gc surviving after exposure to the antimicrobial divided by the percent of Gc surviving in vehicle, and normalized to the vehicle control (= 100%).

Human Lysozyme: Human lysozyme was prepared as in [22]. Gc was incubated with lysozyme for 3 hr unless otherwise indicated. Lysozyme with inactivated hydrolase activity was prepared by boiling as in [22]. Ethylenediaminetetraacetic acid (EDTA) treatment with lysozyme was performed as previously described [22] where the final concentration of Gc with EDTA and lysozyme was 5x10^7^ CFU/mL.

For lysozyme pretreatment with recombinant proteins, vehicle (PBS), lysozyme alone (3 μg/mL), lysozyme + r1063 (3 μg/mL lysozyme + 1.5 μg/mL r1063), lysozyme + rMIP [25, 59] (3 μg/mL lysozyme + 1.5 μg/mL rMIP), and lysozyme + r1981 (3 μg/mL lysozyme + 1.9 μg/mL r1981) were pretreated for 20 min at 37°C. Because r1063 was solubilized in a PBS buffer with SDS, SDS was also added for an equivalent final concentration for all conditions. Gc were exposed to pretreated samples for 5 hr at the following final concentrations: vehicle (PBS), lysozyme + r1063 (1.5 μg/mL lysozyme + 0.75 μg/mL r1063), lysozyme + rMIP (1.5 μg/mL lysozyme + 0.75 μg/mL rMIP), and lysozyme + r1981 (3 μg/mL lysozyme + 0.97 μg/mL r1981).

For mixed Gc experiments, *Δ1981Δ1063* mutant Gc was mixed with *Δ1981Δ1063*::*1981*(WT)-FLAG complemented Gc at a ratio of 1:1 for a total of 5 x 10^5^ CFU/mL final. Gc were incubated together or separately for 1 hr prior to exposure to lysozyme for 3 hr. In mixed infection, *Δ1981Δ1063* mutant Gc was differentiated from *Δ1981Δ1063*::*1981*(WT)-FLAG based on chloramphenicol resistance.

LL-37: For the LL-37 antimicrobial assay, Gc was incubated with LL-37 (from Dr. William Shafer, Emory University) as described [22]. For LL-37 pretreatment, 5x10^5^ CFU/mL Gc was pretreated with 0.4 μg/mL LL-37 for 25 min at 37°C. The bacteria were centrifuged, supernatant removed, and the bacterial pellet resuspended in equivalent volume of 0.5X GCBL, prior to exposure to human lysozyme for 3 hr.

Chicken Egg White lysozyme: Chicken Egg White lysozyme (Sigma) was reconstituted in 10 mM Tris-HCl, pH 8.0, and incubated with Gc for 3 hr at 1,000 μg/mL.

Mutanolysin: Gc was incubated with mutanolysin (Sigma) as previously described [22].

Human Secretions: Pooled human tears and pooled human saliva were pretreated with vehicle or r1981 exactly as in the *M*. *luteus* assay above. Gc was incubated with untreated or treated secretions for 3 hr at the indicated final concentration.

### Antibiotic susceptibility

Gc was exposed to vancomycin Etests (bioMérieux) as described [22] except that Gc was first grown to mid-logarithmic phase in liquid culture, and, if needed, induced with IPTG or AT for 5 hr prior to testing.

### Immunoprecipitation assay

Mid-logarithmic phase Gc (7.5x10^8^ CFU of *Δ1981Δ1063*, *ΔltgA*::*ltgA*-FLAG, *ΔltgD*::*ltgD*-FLAG, *Δ1981Δ1063*::*1981*(WT)-FLAG, *Δ1981Δ1063*::*1063*(WT)-FLAG, and *Δ1981Δ1063*::*1063*(S83A)-FLAG, *Δ1981Δ1063*::*1981*(K103A)-FLAG) in 0.5X GCBL with IPTG were exposed to vehicle or 1,000 μg/mL human lysozyme for 3 hr at 37°C with 5% CO_2_. Gc was subsequently centrifuged, washed once with PBS, and resuspended in 1 mL ice cold lysis buffer (1% v/v Triton-X-100, 150mM NaCl, 2mM EDTA, 20mM Tris-HCl pH 7.5, 1X v/v protease inhibitors (Calbiochem Set V)) for 30 min with end-over-end rotation at 4°C. Insoluble debris and unlysed Gc were pelleted at 10,000xg for 15 min at 4°C. The supernatant (“whole cell lysate”) was removed and incubated with lysis buffer-equilibrated M2 FLAG Affinity Gel (Sigma) for 2 hr with end-over-end rotation at 4°C. The resin was washed six times with ice cold wash buffer (1% v/v Triton-X-100, 150mM NaCl, 2mM EDTA, 20mM Tris-HCl pH 7.5), and subsequently resuspended in 1X sample buffer. Western blots were probed with anti-lysozyme (Abcam 108508), stripped (200mM glycine, 0.1% w/v SDS, 1% v/v Tween-20), and reprobed with anti-FLAG (M2, Sigma).

### Induction of protein expression by lysozyme

For sublethal conditions of lysozyme, 7.5x10^7^ CFU/mL (as opposed to 5x10^5^ CFU/mL used for antimicrobial assays) of Gc, which had been grown to mid-logarithmic phase, was exposed to vehicle or increasing concentrations of human lysozyme in 0.5X GCBL at 37°C with 5% CO_2_ (v/v) for 3 hr. Gc survival was assessed at 0 hr and 3 hr under these conditions, as described above. Otherwise, Gc exposed to vehicle or lysozyme were centrifuged, supernatant removed, and pellet resuspended in sample buffer (12 mM Tris-HCl pH 6.8, 0.4% w/v SDS, 5% v/v Glycerol) without β-mercaptoethanol (BME) or bromophenol blue. Protein concentration was determined by BCA^™^ Protein Assay, followed by addition of BME and bromophenol blue. 5 μg of total protein was separated by SDS-PAGE. Rabbit anti-r1981 antisera and mouse anti-FLAG antibody (M2, Sigma) were used to visualize native Ng_1981 (from WT Gc) and Ng_1063-FLAG (from Gc with *1063*(WT)-FLAG at its native locus) protein, respectively. Blots were stripped and re-probed with rabbit anti-Zwf antibody to confirm equivalent loading. ImageJ was used to measure the percent area for each protein band. The relative band density at each concentration of lysozyme was determined by dividing the vehicle-normalized band density of Ng_1981/Ng_1063-FLAG protein by the vehicle-normalized band density of Zwf protein. Vehicle-normalized band densities were determined by dividing the band density of Ng_1981/Ng_1063-FLAG/Zwf at one concentration of lysozyme by the band density for the corresponding vehicle-treated sample.

### Gc survival after exposure to interleukin 8-treated, adherent primary human neutrophils

Neutrophils were isolated from the venous blood of healthy human subjects as described [60], and used within 2 hr of isolation. Neutrophils were adhered to plastic coverslips in the presence of 10 nM human Interleukin 8 (R&D) in Roswell Park Memorial Institute 1640 medium (RPMI) with 10% v/v FBS at 37°C with 5% CO_2_ (v/v) for at least 30 min prior to infection. Mid-logarithmic phase Gc at a multiplicity of infection of 1 was exposed to neutrophils in a synchronous manner, as previously described [60].

### 1063-FLAG and 1981-FLAG protein expression by imaging flow cytometry

Gc (WT, *Δ1063*::*1063*(WT)-FLAG, and *Δ1981*::*1981*(WT)-FLAG) was grown to mid-logarithmic phase in the presence of IPTG to induce protein expression. Gc (7.5x10^7^ CFU) was centrifuged, resuspended in 30 μg/mL 5-(and-6)-carboxylfluorescein diacetate, succinimidyl ester (CFSE) in Dulbecco’s phosphate-buffered saline (DPBS) + 5 mM MgSO_4_, and incubated for 15 min at 37°C. Bacteria were washed once with DPBS + 5 mM MgSO_4_ and subsequently resuspended in RPMI (no phenol red) with 10% v/v FBS for 10 min at 37°C. Bacteria were centrifuged, resuspended in 8 μg/mL anti-FLAG (M2, sigma) in 100 μL RPMI (no phenol red) with 10% v/v FBS, and incubated for 30 min at 37°C. Bacteria were washed twice with RPMI with 10% v/v FBS, resuspended in 100 μL of 1:400 goat anti-mouse coupled to Alexa Fluor 647 (Thermo) in RPMI (no phenol red) with 10% v/v FBS, and incubated for 30 min at 37°C. Bacteria were washed twice with RPMI with 10% v/v FBS and resuspeded in 2% w/v paraformaldehyde. Data were acquired with an ImageStream^X^ Mark II cytometer operated by INSPIRE software (Amnis) and analyzed using IDEAS Application v6.2 software (Amnis). For analysis, focused cells were gated as determined by a high gradient root mean square for image sharpness. From the focused cells, single cells were gated as defined by a high intensity of CFSE and low side scatter. Focused, single cells were used for analysis of intensity of anti-FLAG/AlexaFluor647.

### Immunofluorescence of 1063-FLAG and 1981-FLAG

1 mL of induced, mid-logarithmic phase Gc (*Δ1063*:*1063*(WT)-FLAG and *Δ1981*:*1981*(WT)-FLAG) was centrifuged and processed as described [37]. For cells that were not permeabilized, PBS with 5% v/v normal goat serum was used as a blocking agent. For FLAG staining, the anti-FLAG antibody was used at 27 μg/mL in PBS with 5% v/v normal goat serum, and goat anti-mouse coupled to Alexa Fluor 647 (Thermo) was used at 1:400 in PBS with 5% v/v normal goat serum. For 1981 staining with antisera, rabbit anti-r1981 antisera was diluted 1:100 in PBS with 5% v/v normal goat serum, and goat anti-rabbit coupled to Alexa Fluor 647 (Thermo) was used at 1:400 in PBS with 5% v/v normal goat serum. Bacterial DNA was stained with 18 μM DAPI (Sigma). Cells were visualized using a Nikon E800 with Hamamatsu Orca-ER camera using Nikon Elements software and processed using Adobe Photoshop CS3.

### Ng_1063 and Ng_1981 protein in bacterial supernatants

Using *1063*(WT)-FLAG native Gc, 7.5x10^7^ CFU of mid-logarithmic phase Gc was centrifuged and resuspended in 1 mL Hanks’ balanced salt solution (HBSS) with 10 mM 4-(2-hydroxyethyl)-1-piperazineethanesulfonic acid and 5 mM sodium bicarbonate. Bacteria were incubated at 37°C with 5% CO_2_ (v/v) for 3 hr and subsequently centrifuged. Supernatants were collected from the bacterial pellet, passed through a 0.2 μm filter, concentrated to a ~60 μL volume using a 3 kDa centrifugal filter unit (Amicon), and brought up to a final volume of 100 μL with sample buffer. Bacterial pellets were washed once with 1X PBS and subsequently lysed (“whole cell lysates”) with 100 μL sample buffer. Equivalent volumes were loaded in an SDS-PAGE gel, and gel transferred for Western blot analysis. Blots were probed with rabbit anti-r1981 antisera to detect native Ng_1981 protein, and then stripped and reprobed with mouse anti-FLAG (M2, sigma) to detect native Ng_1063 protein. Blots were also probed with rabbit anti-Zwf (Aleksandra Sikora, Oregon State University [61]) as a cell lysate loading control.

### Statistics

Experimental values presented display the mean ± the standard error of the mean (SEM) of at least three independent replicates. For experiments using neutrophils, at least 3 independent donors were used. Unless otherwise indicated, a two tailed student’s *t*-test was performed, and significance was determined as a *p*-value less than 0.05.

## Supporting information

S1 Fig Ng_1981 is important for Gc resistance to lysozyme. A. MUSCLE alignment for MS11 Ng_1063 (orange) with Ng_1981 (signal sequences underlined). Asterisks (*) denote positions in the sequence with a fully conserved residue. Colons (:) and periods (.) denote amino acids with strongly or weakly similar properties, respectively. S83 and K103 residues of Ng_1063 are highlighted in a magenta and red box, respectively. B. WT and *ΔltgAΔltgD* Gc were exposed to 1.5 μg/mL human lysozyme (HL), which had been pretreated with recombinant 1981 (r1981, 0.97 μg/mL final in assay), for 5 hr. WT and *ΔltgAΔltgD* data for vehicle control and lysozyme alone are from Fig 2B. Gc survival was determined as in Fig 2B. *n* = 6–15 biological replicates. C. WT, *ΔltgAΔltgD*, and *ΔltgAΔltgD*::*1981+* complement were exposed to 10 μg/mL human lysozyme (HL) for 3 hr. Gc survival was determined as in Fig 2B. WT and *ΔltgAΔltgD* data are from Fig 2C. *n* = 3–6 biological replicates. D. *Δ1981* Gc complemented with *1981*(WT)-FLAG, *1981*(S76A)-FLAG, or *1981*(K99A)-FLAG were grown to mid-log phase with 1mM IPTG as in Fig 5D. Bacterial lysates were separated by SDS-PAGE and immunoblotted using anti-FLAG antibody. Blots were also probed with anti-Zwf antibody as a loading control. Shown are 3 biological replicates for each strain. E. WT, *Δ1981*, *Δ1981*::*1981*(WT)-FLAG, *Δ1981*::*1981*(S76A)-FLAG, and *Δ1981*::*1981*(K99A)-FLAG complement Gc were exposed to 1,000 μg/mL human lysozyme (HL) for 3 hr. Gc survival was determined as in Fig 2B. *n* = 4–17 biological replicates. All values are represented as the mean ± SEM. *p < 0.05; two tailed *t*-test.
(PDF)

S2 Fig Contribution of Ng_1063 to Gc survival under membrane-permeable conditions. A. WT and *Δ1063* Gc were exposed to LL-37 for 45 min. Gc survival was determined as in Fig 2B. Values are represented as the mean ± SEM. *n* = 4–5 biological replicates. B. WT and *Δ1063* Gc were exposed to 0.4 μg/mL LL-37 for 25 min and LL-37 subsequently removed, prior to exposure to human lysozyme for 3 hr. Gc survival was determined as in Fig 2B. *n* = 6–9 biological replicates. C. WT and *Δ1063* Gc were permeabilized with 1mM EDTA with concomitant exposure to human lysozyme for 30 min. Gc survival was determined as in Fig 2B. *n* = 3 biological replicates. All values are represented as the mean ± SEM. Differences between strains were not statistically significant.
(PDF)

S3 Fig Contribution of Ng_1063 and Ng_1981 to Gc survival in the *ΔltgAΔltgD* mutant background. *ΔltgAΔltgD*, *ΔltgAΔltgDΔ1063*, *ΔltgAΔltgDΔ1981*, and *ΔltgAΔltgDΔ1981Δ1063* Gc were exposed to human lysozyme for 1 hr. Gc survival was determined as in Fig 2B. Values are represented as the mean ± SEM. NS, not significant. *p < 0.05; two tailed *t*-test, *n* = 3–15 biological replicates.
(PDF)

S4 Fig Contribution of Ng_1063 to Gc survival from additional peptidoglycan muramidases. A. MUSCLE alignment of human lysozyme with chicken egg white lysozyme and mutanolysin (signal sequences removed from lysozymes). Asterisks (*) denote positions in the sequence with a fully conserved residue. Colons (:) and periods (.) denote amino acids with strongly or weakly similar properties, respectively. The glutamic acid and aspartic acid active site residues of lysozyme are boxed in yellow and blue, respectively. B. WT, *Δ1981*, *Δ1981*::*1981*+ complement, *Δ1981Δ1063*, and *Δ1981Δ1063*::*1063*+ complement Gc were exposed to chicken egg white lysozyme (CHEWL) for 3 hr. Gc survival was determined as in Fig 2B. *n* = 3–9 biological replicates. C. WT, *Δ1981*, *Δ1981*::*1981*+ complement, *Δ1981Δ1063*, *Δ1981Δ1063*::*1063*+ complement, and *ΔltgAΔltgD* Gc were exposed to mutanolysin for 3 hr. Gc survival was determined as in Fig 2B. NS, not significant. *n* = 3–6 biological replicates. Values are represented as the mean ± SEM. *p < 0.05; two tailed *t*-test.
(PDF)

S5 Fig Expression of 1063(WT)-FLAG, 1063(S83A)-FLAG, and 1063(K103A)-FLAG. *Δ1981Δ1063* Gc complemented with *1063*(WT)-FLAG, *1063*(S83A)-FLAG, and *1063*(K103A)-FLAG were grown to mid-log phase with 1mM IPTG as in Fig 5D. Bacterial lysates were separated by SDS-PAGE and immunoblotted using anti-FLAG antibody. Blots were then stripped and probed with anti-Zwf antibody as a loading control. Shown are 3 biological replicates for each strain.
(PDF)

S6 Fig Lysis of *Micrococcus luteus* upon exposure to human tears and saliva. Pooled and diluted human tears (0.01X) (A) and human saliva (0.05X) (B) were treated with r1981 or vehicle for 20 min at 37°C prior to exposure to *Micrococcus luteus*. Lysis was measured via the decrease in absorbance at 450 nm over time. Shown are representative graphs from 1 of 3 biological replicates.
(PDF)

S7 Fig Expression of Ng_1063 and Ng_1981 does not enhance survival of *ΔltgAΔltgD* Gc from primary human neutrophils. Human neutrophils were exposed to WT, *ΔltgAΔltgD*, *ΔltgAΔltgD*::*1063+* complement, and *ΔltgAΔltgD*::*1981+* complement Gc as in Fig 6C. Values are represented as the mean ± SEM. NS, not significant. *p ≤0.05 for *ΔltgAΔltgD* compared to WT; two tailed *t*-test, *n* = 3–6 independent experiments.
(PDF)

S8 Fig Ng_1981 is not detected on the surface of Gc by immunofluorescence microscopy using anti-r1981 antisera. *Δ1981*::*1981*(WT)-FLAG Gc were processed for immunofluorescence with (+ perm) or without (- perm) methanol and Triton-X-100 permeabilization, as in Fig 7D. Bacterial DNA was stained with DAPI (blue), and 1981-FLAG was detected using antisera raised against r1981 (red). Scale bar, 5μm.
(PDF)

S1 Table Contribution of Ng_1063 and Ng_1981 to the minimum inhibitory concentration (MIC) of vancomycin. WT, *Δ1981*, *Δ1981Δ1063*, *ΔltgAΔltgD*, *ΔltgAΔltgD*::*1063+* complement, and *ΔltgAΔltgD*::*1982+* complement were spread on solid media and exposed to a Vancomycin Etest strip. The MIC for each strain was determined according to the manufacturer’s instructions. *n* = 3 biological replicates.
(PDF)

S2 Table Analysis of NEIS1425 (*ng1063*) alleles and number of isolates per *Neisseria* species. The PubMLST database identified 284 alleles (of which 169 have representative isolates) for *ng1063* in *Neisseria* species, which culminate to make 95 non-redundant proteins. Numbers in parentheses indicate alleles which produce proteins with an exact amino acid sequence match. The most highly represented alleles for *Neisseria meningitidis* and *Neisseria gonorrhoeae* sequenced isolates are highlighted in blue and orange, respectively. The PubMLST database was accessed on December 1, 2017.
(PDF)

S3 Table Alignment of non-redundant NEIS1425 (*ng1063*) alleles from *Neisseria*. Clustal omega alignment of non-redundant *ng1063* alleles (residues 80–110) from *Neisseria* identified in S2 Table. The Serine 83 and Lysine103 residues in red and blue, respectively, are conserved across all species.
(PDF)

S4 Table Strains and plasmids used in this study. (PDF)

S1 Dataset For the reader’s reference, each tab of this Excel spreadsheet shows the CFU/mL calculated from each lysozyme experiment for each time point. (XLSX)
