# Supplementary material for: Neisseria gonorrhoeae employs two protein inhibitors to evade killing by human lysozyme
Source: PLoS Pathog. 2018 Jul 5;14(7):e1007080. doi: 10.1371/journal.ppat.1007080 (PMC6033460; doi:10.1371/journal.ppat.1007080)
Supplement: S1 Fig — A. MUSCLE alignment for MS11 Ng_1063 (orange) with Ng_1981 (signal sequences underlined). Asterisks (*) denote positions in the sequence with a fully conserved residue. Colons (:) and periods (.) denote amino acids with strongly or weakly similar properties, respectively. S83 and K103 residues of Ng_1063 are highlighted in a magenta and red box, respectively. B. WT and ΔltgAΔltgD Gc were exposed to 1.5 μg/mL human lysozyme (HL), which had been pretreated with recombinant 1981 (r1981, 0.97 μg/mL final in assay), for 5 hr. WT and ΔltgAΔltgD data for vehicle control and lysozyme alone are from Fig 2B. Gc survival was determined as in Fig 2B. n = 6–15 biological replicates. C. WT, ΔltgAΔltgD, and ΔltgAΔltgD::1981+ complement were exposed to 10 μg/mL human lysozyme (HL) for 3 hr. Gc survival was determined as in Fig 2B. WT and ΔltgAΔltgD data are from Fig 2C. n = 3–6 biological replicates. D. Δ1981 Gc complemented with 1981(WT)-FLAG, 1981(S76A)-FLAG, or 1981(K99A)-FLAG were grown to mid-log phase with 1mM IPTG as in Fig 5D. Bacterial lysates were separated by SDS-PAGE and immunoblotted using anti-FLAG antibody. Blots were also probed with anti-Zwf antibody as a loading control. Shown are 3 biological replicates for each strain. E. WT, Δ1981, Δ1981::1981(WT)-FLAG, Δ1981::1981(S76A)-FLAG, and Δ1981::1981(K99A)-FLAG complement Gc were exposed to 1,000 μg/mL human lysozyme (HL) for 3 hr. Gc survival was determined as in Fig 2B. n = 4–17 biological replicates. All values are represented as the mean ± SEM. *p < 0.05; two tailed t-test. (PDF) [file ppat.1007080.s001.pdf]

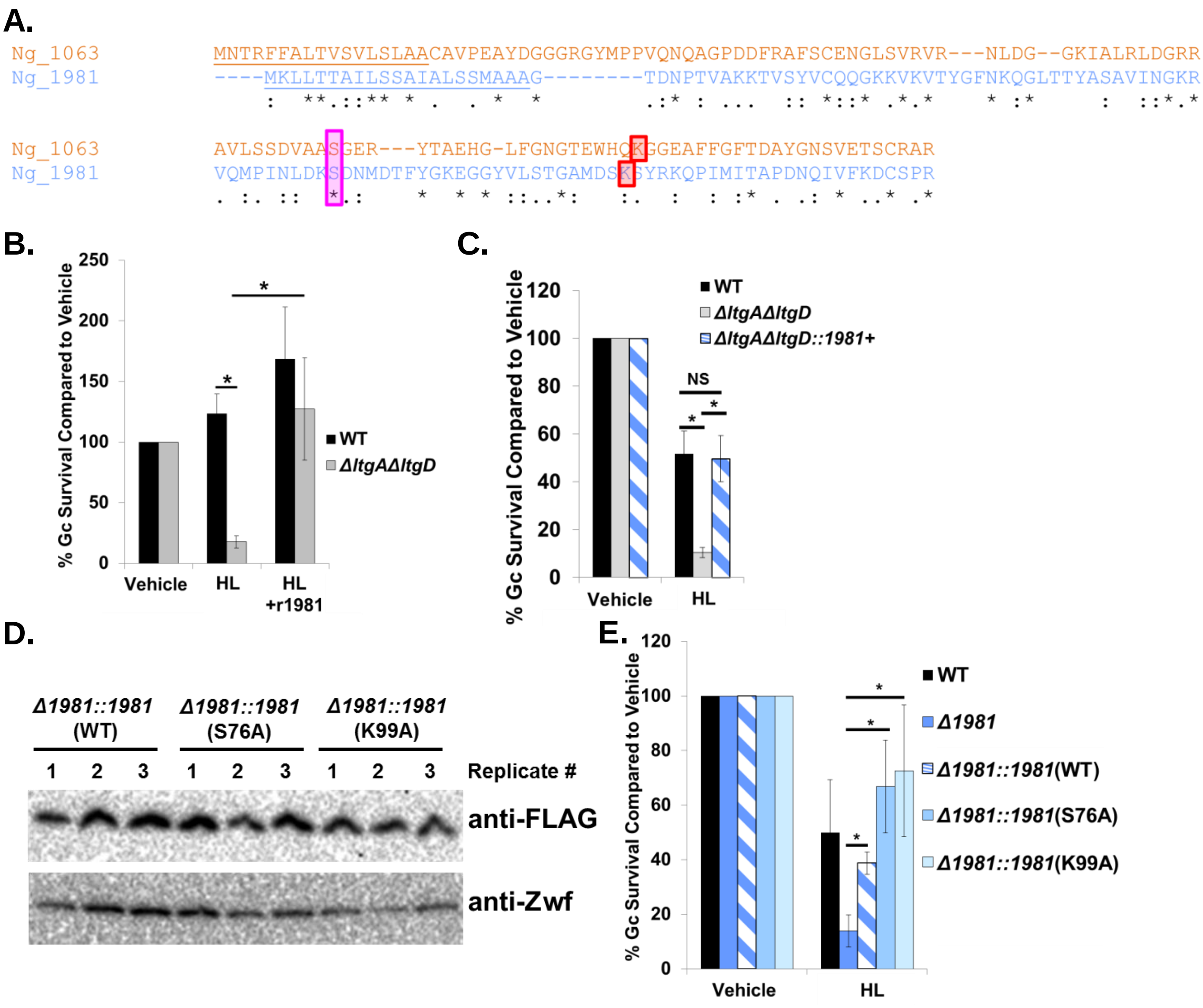

S1 Fig. Ng_1981 is important for Gc resistance to lysozyme.

A. MUSCLE alignment for MS11 Ng_1063 (orange) with Ng_1981 (signal sequences underlined). Asterisks (*) denote positions in the sequence with a fully conserved residue. Colons (:) and periods (.) denote amino acids with strongly or weakly similar properties, respectively. S83 and K103 residues of Ng_1063 are highlighted in a magenta and red box, respectively.

B. WT and *ΔltgAΔltgD* Gc were exposed to 1.5 μg/mL human lysozyme (HL), which had been pretreated with recombinant 1981 (r1981, 0.97 μg/mL final in assay), for 5 hr. WT and *ΔltgAΔltgD* data for vehicle control and lysozyme alone are from Fig. 2B. Gc survival was determined as in Fig. 2B. n = 6-15 biological replicates.

C. WT, *ΔltgAΔltgD*, and *ΔltgAΔltgD::1981+* complement were exposed to 10 μg/mL human lysozyme (HL) for 3 hr. Gc survival was determined as in Fig. 2B. WT and *ΔltgAΔltgD* data are from Fig. 2C. n = 3-6 biological replicates.

D. *Δ1981*complemented with *1981*(WT)-FLAG, *1981*(S76A)-FLAG, and *1981*(K99A)-FLAG were grown to mid-log phase with 1mM IPTG as in Fig. 5D. Bacterial lysates were separated by SDS-PAGE and immunoblotted using anti-FLAG antibody. Blots were also probed with anti-Zwf antibody as a loading control. Shown are 3 biological replicates for each strain.

E. WT, *Δ1981*, *Δ1981::1981*(WT)-FLAG, *Δ1981::1981*(S76A)-FLAG, and *Δ1981::1981*(K99A)-FLAG complement Gc were exposed to 1,000 μg/mL human lysozyme (HL) for 3 hr. Gc survival was determined as in Fig. 2B. n = 4-17 biological replicates.

All values are represented as the mean ± SEM. *$p < 0.05$; two tailed t-test.
