# Supplementary material for: Neisseria gonorrhoeae employs two protein inhibitors to evade killing by human lysozyme
Source: PLoS Pathog. 2018 Jul 5;14(7):e1007080. doi: 10.1371/journal.ppat.1007080 (PMC6033460; doi:10.1371/journal.ppat.1007080)
Supplement: S2 Fig — A. WT and Δ1063 Gc were exposed to LL-37 for 45 min. Gc survival was determined as in Fig 2B. Values are represented as the mean ± SEM. n = 4–5 biological replicates. B. WT and Δ1063 Gc were exposed to 0.4 μg/mL LL-37 for 25 min and LL-37 subsequently removed, prior to exposure to human lysozyme for 3 hr. Gc survival was determined as in Fig 2B. n = 6–9 biological replicates. C. WT and Δ1063 Gc were permeabilized with 1mM EDTA with concomitant exposure to human lysozyme for 30 min. Gc survival was determined as in Fig 2B. n = 3 biological replicates. All values are represented as the mean ± SEM. Differences between strains were not statistically significant. (PDF) [file ppat.1007080.s002.pdf]

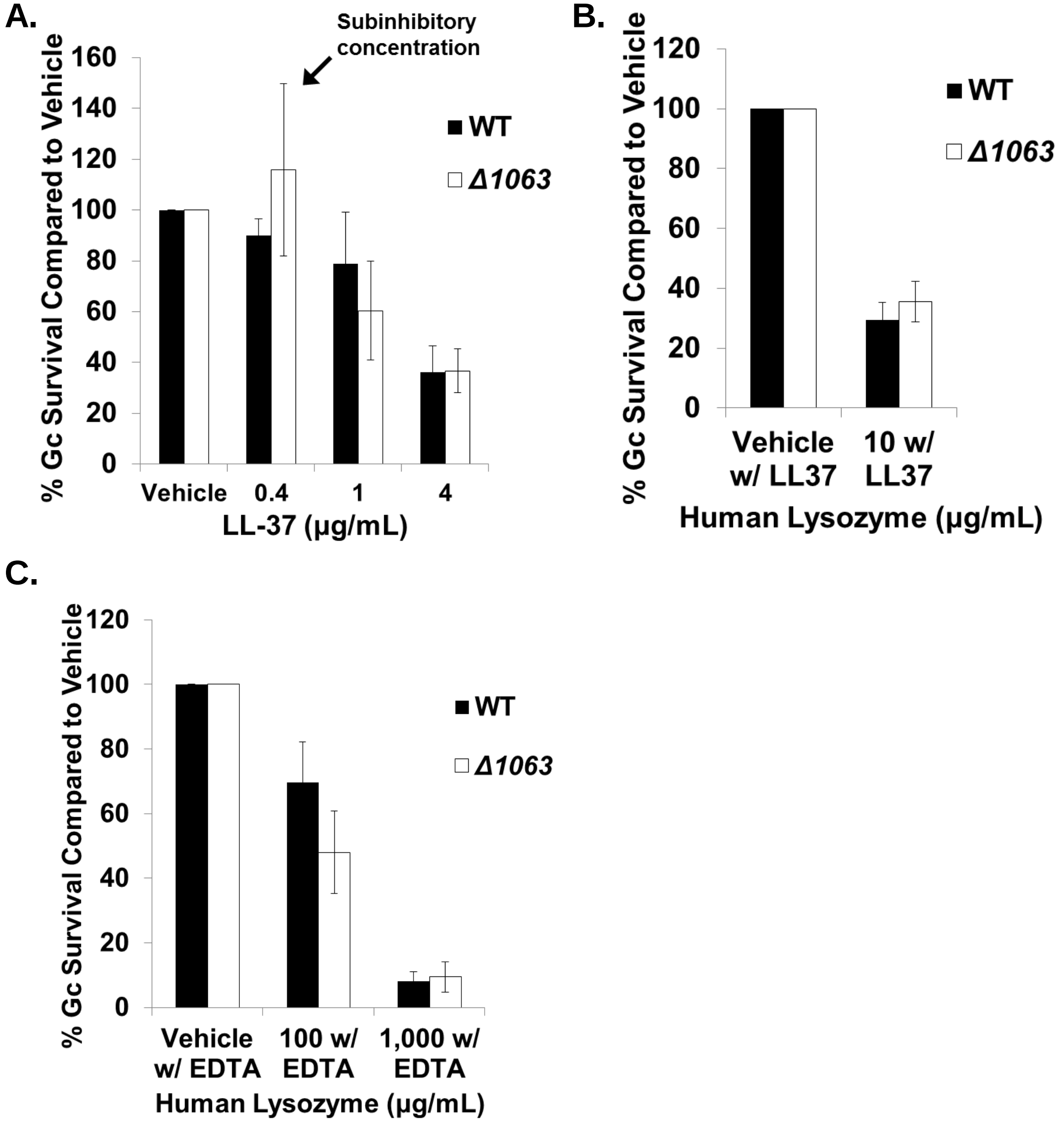

S2 Fig. Contribution of Ng_1063 to Gc survival under membrane-permeable conditions.

A. WT and *Δ1063* Gc were exposed to LL-37 for 45 min. Gc survival was determined as in Fig. 2B. Values are represented as the mean ± SEM. *n* = 4-5 biological replicates.

B. WT and *Δ1063* Gc were exposed to 0.4 µg/mL LL-37 for 25 min and LL-37 subsequently removed, prior to exposure to human lysozyme for 3 hr. Gc survival was determined as in Fig. 2B. *n* = 6-9 biological replicates.

C. WT and *Δ1063* Gc were permeabilized with 1mM EDTA with concomitant exposure to human lysozyme for 30 min. Gc survival was determined as in Fig. 2B. *n* = 3 biological replicates.

All values are represented as the mean ± SEM. Differences between strains were not statistically significant.
