# Supplementary material for: Neisseria gonorrhoeae employs two protein inhibitors to evade killing by human lysozyme
Source: PLoS Pathog. 2018 Jul 5;14(7):e1007080. doi: 10.1371/journal.ppat.1007080 (PMC6033460; doi:10.1371/journal.ppat.1007080)
Supplement: S3 Fig — ΔltgAΔltgD, ΔltgAΔltgDΔ1063, ΔltgAΔltgDΔ1981, and ΔltgAΔltgDΔ1981Δ1063 Gc were exposed to human lysozyme for 1 hr. Gc survival was determined as in Fig 2B. Values are represented as the mean ± SEM. NS, not significant. *p < 0.05; two tailed t-test, n = 3–15 biological replicates. (PDF) [file ppat.1007080.s003.pdf]

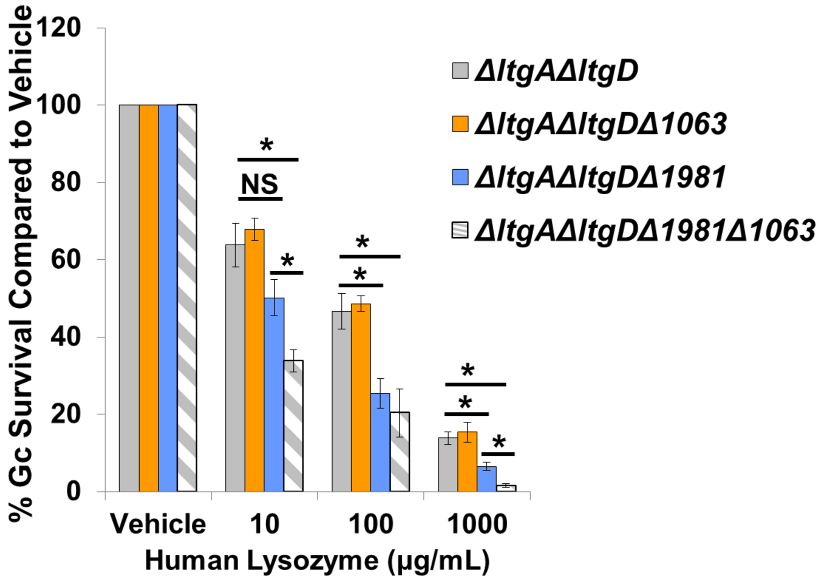

S3 Fig. Contribution of Ng_1063 and Ng_1981 to Gc survival in the *ΔltgAΔltgD* mutant background.
ΔltgAΔltgD, ΔltgAΔltgDΔ1063, ΔltgAΔltgDΔ1981, and *ΔltgAΔltgDΔ1981Δ1063* Gc were exposed to human lysozyme for 1 hr. Gc survival was determined as in Fig. 2B. Values are represented as the mean ± SEM. NS, not significant. $^{*}p < 0.05$; two tailed *t*-test, n = 3-15 biological replicates.
