# Supplementary material for: Neisseria gonorrhoeae employs two protein inhibitors to evade killing by human lysozyme
Source: PLoS Pathog. 2018 Jul 5;14(7):e1007080. doi: 10.1371/journal.ppat.1007080 (PMC6033460; doi:10.1371/journal.ppat.1007080)
Supplement: S4 Fig — A. MUSCLE alignment of human lysozyme with chicken egg white lysozyme and mutanolysin (signal sequences removed from lysozymes). Asterisks (*) denote positions in the sequence with a fully conserved residue. Colons (:) and periods (.) denote amino acids with strongly or weakly similar properties, respectively. The glutamic acid and aspartic acid active site residues of lysozyme are boxed in yellow and blue, respectively. B. WT, Δ1981, Δ1981::1981+ complement, Δ1981Δ1063, and Δ1981Δ1063::1063+ complement Gc were exposed to chicken egg white lysozyme (CHEWL) for 3 hr. Gc survival was determined as in Fig 2B. n = 3–9 biological replicates. C. WT, Δ1981, Δ1981::1981+ complement, Δ1981Δ1063, Δ1981Δ1063::1063+ complement, and ΔltgAΔltgD Gc were exposed to mutanolysin for 3 hr. Gc survival was determined as in Fig 2B. NS, not significant. n = 3–6 biological replicates. Values are represented as the mean ± SEM. *p < 0.05; two tailed t-test. (PDF) [file ppat.1007080.s004.pdf]

A.

```
human lysozyme   KVFERCELARTLKRLGMDGYRGISLANWMCLAKWESGYN----------TRATNYNAGDR
chicken lysozyme KVFGRCELAAAMKRHGLDNYRGYSLGNWVCAAKFESNFN----------TQATNRNTDG-
mutanolysin      ---------------DTSGVQGIDVSHWQGSINWSSVKSAGMSFAYIKATEGTNYKDDRF
                                . .. .* .:.:*    ::.*  .          * .** : .

human lysozyme   STDY------GIFQINSRY---------------------WCNDGKT-PGAVNACHLS--
chicken lysozyme STDY------GILQINSRW---------------------WCNDGRT-PGSRNLCNIP--
mutanolysin      SANYTNAYNAGIIRGAYHFARPNASSGTAQADYFASNGGGWSRDNRTLPGVLDIEHNPSG
                 *::*      **:.   .:                     *..*..* **  :  : .

human lysozyme   --CSALLQDNIADAVA-----CAKRVVRD-------------------------------
chicken lysozyme --CSALLSSDITASVN-----CAKKIVSD-------------------------------
mutanolysin      AMCYGLSTTQMRTWINDFHARYKARTTRDVVIYTTASWWNTCTGSWNGMAAKSPFWVAHW
                   * .*   ::   :         . . *

human lysozyme   -------PQGIRAWVAWRNRCQNR------DV-RQYVQGCGV----------
chicken lysozyme -------GNGMNAWVAWRNRCKGT------DV-QAWIRGCRL----------
mutanolysin      GVSAPTVPSGFPTWTFWQYSATGRVGGVSGDVDRNKFNGSAARLLALANNTA
                         .*: :*. *.  . .       ** .  ..*.
```

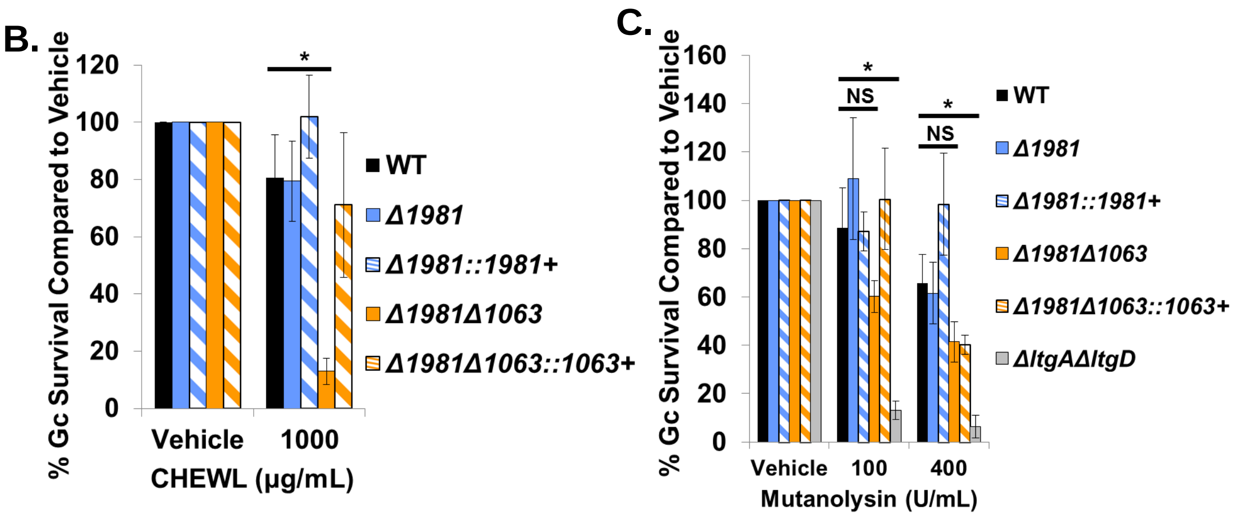

S4 Fig. Contribution of Ng_1063 to Gc survival from additional peptidoglycan muramidases.

A. MUSCLE alignment of human lysozyme with chicken egg white lysozyme and mutanolysin (signal sequences removed from lysozymes). Asterisks (*****) denote positions in the sequence with a fully conserved residue. Colons (**:**) and periods (**.**) denote amino acids with strongly or weakly similar properties, respectively. The glutamic acid and aspartic acid active site residues of lysozyme are boxed in yellow and blue, respectively.

B. WT, *Δ1981*, *Δ1981::1981+* complement, *Δ1981Δ1063*, and *Δ1981Δ1063::1063+* complement Gc were exposed to chicken egg white lysozyme (CHEWL) for 3 hr. Gc survival was determined as in Fig. 2B. n = 3-9 biological replicates.

C. WT, *Δ1981*, *Δ1981::1981+* complement, *Δ1981Δ1063*, *Δ1981Δ1063::1063+* complement, and *ΔltgAΔltgD* Gc were exposed to mutanolysin for 3 hr. Gc survival was determined as in Fig. 2B. NS, not significant. n = 3-6 biological replicates.

Values are represented as the mean ± SEM. *$p < 0.05$; two tailed t-test.
