# Supplementary material for: Neisseria gonorrhoeae employs two protein inhibitors to evade killing by human lysozyme
Source: PLoS Pathog. 2018 Jul 5;14(7):e1007080. doi: 10.1371/journal.ppat.1007080 (PMC6033460; doi:10.1371/journal.ppat.1007080)
Supplement: S5 Fig — Δ1981Δ1063 Gc complemented with 1063(WT)-FLAG, 1063(S83A)-FLAG, and 1063(K103A)-FLAG were grown to mid-log phase with 1mM IPTG as in Fig 5D. Bacterial lysates were separated by SDS-PAGE and immunoblotted using anti-FLAG antibody. Blots were then stripped and probed with anti-Zwf antibody as a loading control. Shown are 3 biological replicates for each strain. (PDF) [file ppat.1007080.s005.pdf]

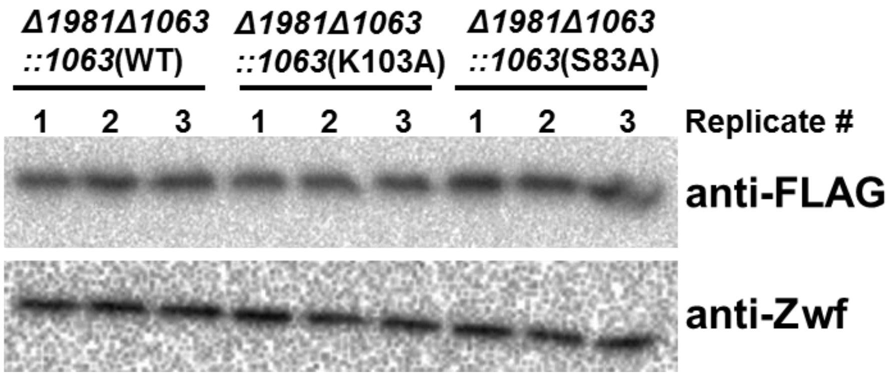

S5 Fig. Expression of 1063(WT)-FLAG, 1063(S83A)-FLAG, and 1063(K103A)-FLAG.

Δ1981Δ1063 Gc complemented with *1063*(WT)-FLAG, *1063*(S83A)-FLAG, and *1063*(K103A)-FLAG were grown to mid-log phase with 1mM IPTG as in Fig. 5D. Bacterial lysates were separated by SDS-PAGE and immunoblotted using anti-FLAG antibody. Blots were then stripped and probed with anti-Zwf antibody as a loading control. Shown are 3 biological replicates for each strain.
