# Supplementary material for: Neisseria gonorrhoeae employs two protein inhibitors to evade killing by human lysozyme
Source: PLoS Pathog. 2018 Jul 5;14(7):e1007080. doi: 10.1371/journal.ppat.1007080 (PMC6033460; doi:10.1371/journal.ppat.1007080)
Supplement: S6 Fig — Pooled and diluted human tears (0.01X) (A) and human saliva (0.05X) (B) were treated with r1981 or vehicle for 20 min at 37°C prior to exposure to Micrococcus luteus. Lysis was measured via the decrease in absorbance at 450 nm over time. Shown are representative graphs from 1 of 3 biological replicates. (PDF) [file ppat.1007080.s006.pdf]

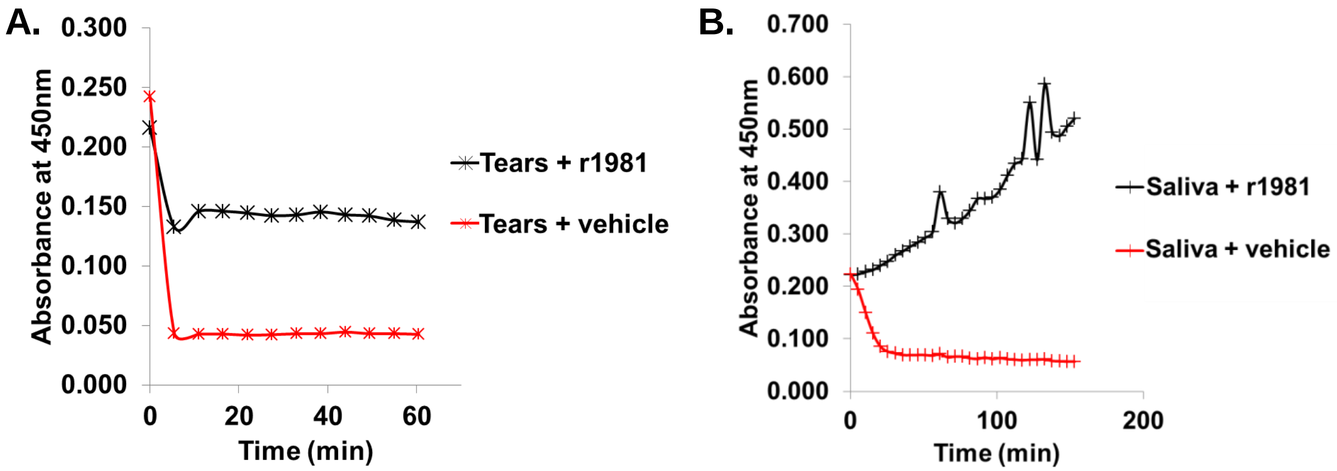

S6 Fig. Lysis of *Micrococcus luteus* upon exposure to human tears and saliva.
Pooled and diluted human tears (0.01X) (A) and human saliva (0.05X) (B) were treated with r1981 or vehicle for 20 min at 37°C prior to exposure to *Micrococcus luteus*. Lysis was measured via the decrease in absorbance at 450 nm over time. Shown are representative graphs from 1 of 3 biological replicates.
