# Supplementary material for: Neisseria gonorrhoeae employs two protein inhibitors to evade killing by human lysozyme
Source: PLoS Pathog. 2018 Jul 5;14(7):e1007080. doi: 10.1371/journal.ppat.1007080 (PMC6033460; doi:10.1371/journal.ppat.1007080)
Supplement: S7 Fig — Human neutrophils were exposed to WT, ΔltgAΔltgD, ΔltgAΔltgD::1063+ complement, and ΔltgAΔltgD::1981+ complement Gc as in Fig 6C. Values are represented as the mean ± SEM. NS, not significant. *p ≤0.05 for ΔltgAΔltgD compared to WT; two tailed t-test, n = 3–6 independent experiments. (PDF) [file ppat.1007080.s007.pdf]

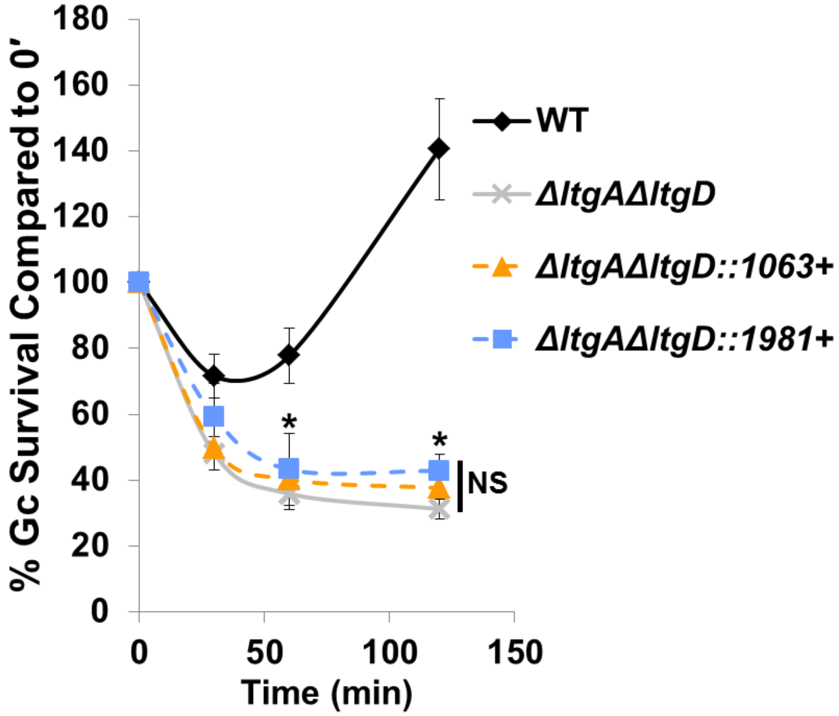

S7 Fig. Expression of Ng_1063 and Ng_1981 does not enhance survival of *ΔltgAΔltgD* Gc from primary human neutrophils.

Human neutrophils were exposed to WT, *ΔltgAΔltgD*, *ΔltgAΔltgD::1063+* complement, and *ΔltgAΔltgD::1981+* complement Gc as in Fig. 6C. Values are represented as the mean ± SEM. NS, not significant. *$p \leq 0.05$ for *ΔltgAΔltgD* compared to WT; two tailed *t*-test, n = 3-6 independent experiments.
