# Supplementary material for: Neisseria gonorrhoeae employs two protein inhibitors to evade killing by human lysozyme
Source: PLoS Pathog. 2018 Jul 5;14(7):e1007080. doi: 10.1371/journal.ppat.1007080 (PMC6033460; doi:10.1371/journal.ppat.1007080)
Supplement: S8 Fig — Δ1981::1981(WT)-FLAG Gc were processed for immunofluorescence with (+ perm) or without (- perm) methanol and Triton-X-100 permeabilization, as in Fig 7D. Bacterial DNA was stained with DAPI (blue), and 1981-FLAG was detected using antisera raised against r1981 (red). Scale bar, 5μm. (PDF) [file ppat.1007080.s008.pdf]

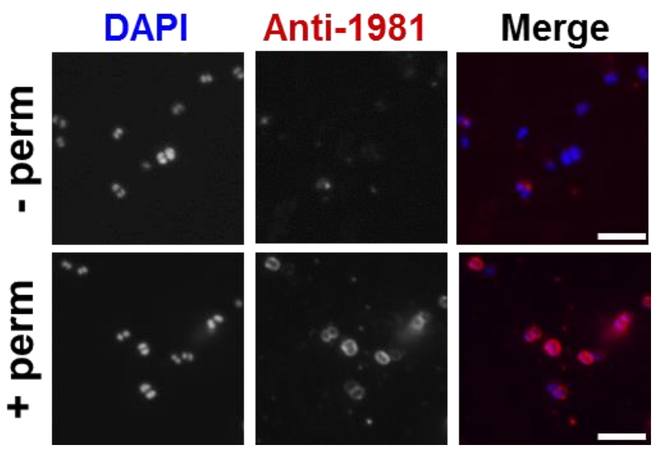

S8 Fig. Ng_1981 is not detected on the surface of Gc by immunofluorescence microscopy using anti-r1981 antisera.
Δ1981::1981(WT)-FLAG Gc were processed for immunofluorescence with (+ perm) or without (-perm) methanol and Triton-X-100 permeabilization, as in Fig. 7D. Bacterial DNA was stained with DAPI (blue), and 1981-FLAG was detected using antisera raised against r1981 (red). Scale bar, 5μm.
