# Supplementary material for: Neisseria gonorrhoeae employs two protein inhibitors to evade killing by human lysozyme
Source: PLoS Pathog. 2018 Jul 5;14(7):e1007080. doi: 10.1371/journal.ppat.1007080 (PMC6033460; doi:10.1371/journal.ppat.1007080)
Supplement: S1 Table — WT, Δ1981, Δ1981Δ1063, ΔltgAΔltgD, ΔltgAΔltgD::1063+ complement, and ΔltgAΔltgD::1982+ complement were spread on solid media and exposed to a Vancomycin Etest strip. The MIC for each strain was determined according to the manufacturer’s instructions. n = 3 biological replicates. (PDF) [file ppat.1007080.s009.pdf]

Strain	MIC for Vancomycin (μg/mL)
WT	16-24
Δ1981	16-24
Δ1981Δ1063	16-24
ΔltgAΔltgD	8
ΔltgAΔltgD::1063+	4-8
ΔltgAΔltgD::1981+	6-8

S1 Table. Contribution of Ng_1063 and Ng_1981 to the minimum inhibitory concentration (MIC) of vancomycin.
WT, *Δ1981*, *Δ1981Δ1063*, *ΔltgAΔltgD*, *ΔltgAΔltgD::1063*+ complement, and *ΔltgAΔltgD::1982*+ complement were spread on solid media and exposed to a Vancomycin Etest strip. The MIC for each strain was determined according to the manufacturer's instructions. $n = 3$ biological replicates.
