# Supplementary material for: Neisseria gonorrhoeae employs two protein inhibitors to evade killing by human lysozyme
Source: PLoS Pathog. 2018 Jul 5;14(7):e1007080. doi: 10.1371/journal.ppat.1007080 (PMC6033460; doi:10.1371/journal.ppat.1007080)
Supplement: S2 Table — The PubMLST database identified 284 alleles (of which 169 have representative isolates) for ng1063 in Neisseria species, which culminate to make 95 non-redundant proteins. Numbers in parentheses indicate alleles which produce proteins with an exact amino acid sequence match. The most highly represented alleles for Neisseria meningitidis and Neisseria gonorrhoeae sequenced isolates are highlighted in blue and orange, respectively. The PubMLST database was accessed on December 1, 2017. (PDF) [file ppat.1007080.s010.pdf]

**S2 Table. Analysis of NEIS1425 (*ng1063*) alleles and number of isolates per *Neisseria* species.** The PubMLST database identified 284 alleles (of which 169 have representative isolates) for *ng1063* in *Neisseria* species, which culminate to make 95 non-redundant proteins. Numbers in parentheses indicate alleles which produce proteins with an exact amino acid sequence match. The most highly represented alleles for *Neisseria meningitidis* and *Neisseria gonorrhoeae* sequenced isolates are highlighted in blue and orange, respectively. The PubMLST database was accessed on December 1, 2017.

| Allele (NEIS1425, *ngo1063*) | Pathogenic *Neisseria* species | | Commensal *Neisseria* species | | | | Total |
|---|---|---|---|---|---|---|---|
| | ***N. meningitidis*** | ***N. gonorrhoeae*** | ***N. lactamica*** | ***N. polysaccharea*** | ***N. cinerea*** | ***N. bergeri*** | |
| **1** (69, 100, 179, 251) | 2375 | | | | | | **2375** |
| **2** (3, 5, 68, 70, 76, 124, 135,138) | 1146 | | | | | | **1146** |
| **4** (9, 21, 24, 38, 44, 86, 110, 111, 127, 128, 172, 173, 174) | 1755 | | | | | | **1755** |
| **6** (59, 136, 230) | 625 | | | | | | **625** |
| **7** (10, 23, 63, 106, 121, 160, 166, 215) | 1196 | | | | | | **1196** |
| **8** | 1 | | | | | | **1** |
| **11** | 93 | | | | | | **93** |
| **12** (45, 51, 58, 81, 122, 167, 231) | 638 | | | | | | **638** |
| **13** (52, 71, 96, 130, 165, 278) | 334 | | | | | | **334** |
| **14** (17, 54, 85, 89,268) | 293 | | | | | | **293** |
| **15** | 959 | | | | | | **959** |
| **16** | 191 | | | | | | **191** |
| **18** | 3 | | | | | | **3** |
| **19** (56, 57, 141) | 117 | | | | | | **117** |
| **20** | | | | 3 | | | **3** |
| **22** | 53 | | | | | | **53** |
| **25** (46) | 3 | | 98 | | | | **101** |
| **29** (116, 118, 153, 154) | | 3681 | | | | | **3681** |
| **30** (187) | 2 | | 1 | | | | **3** |
| **31** (75) | 1 | | 2 | | 1 | | **4** |
| **32** (48) | 1 | | 6 | | | | **7** |
| **33** | 1 | | | | | 1 | **2** |
| **34** | | | | 4 | | | **4** |
| **35** | | | | 1 | | | **1** |
| **36** | | | | | 1 | | **1** |
| **37** | 24 | | | | | | **24** |
| **39** (281) | 3 | | | | | | **3** |
| **40** | 1 | | | | | | **1** |
| **41** | 1 | | | | | | **1** |
| **42** | 1 | | | | | | **1** |
| **43** | 5 | | | | | | **5** |
| **47** | | | 2 | | | | **2** |
| **49** | | | 1 | | | | **1** |
| **50** | | 79 | | | | | **79** |
| **53** | 2 | | | | | | **2** |
| **55** | 2 | | | | | | **2** |
| **60** | 4 | | | | | | **4** |
| **61** | 1 | | | | | | **1** |
| **62** | 1 | | | | | | **1** |
| **64** (66, 176) | 4 | | | | | | **4** |
| **65** | 2 | | | | | | **2** |
| **67** (102) | 10 | | | | | | **10** |
| **72** | 1 | | | | | | **1** |
| **73** | 7 | | | | | | **7** |
| **74** | 1 | | | | | | **1** |
| **77** | 3 | | | | | | **3** |
| **78** (107, 284) | | | 25 | 3 | | | **28** |
| **79** (145) | 1 | | | 1 | | | **2** |
| **80** | 1 | | | | | | **1** |
| **82** | 1 | | | | | | **1** |
| **83** | 1 | | | | | | **1** |
| **84** | 1 | | | | | | **1** |
| **88** | 2 | | | | | | **2** |
| **90** | 1 | | | | | | **1** |
| **91** | | | 2 | 2 | | | **4** |
| **92** | | | 1 | | | | **1** |
| **103** | 3 | | | | | | **3** |
| **104** (125) | 2 | | | | | | **2** |
| **108** | 1 | | | | | | **1** |
| **117** | | 1 | | | | | **1** |
| **119** | 1 | | | | | | **1** |
| **120** (142) | 4 | | | | | | **4** |

| Allele (NEIS1425, *ngo1063*) | Pathogenic *Neisseria* species | | Commensal *Neisseria* species | | | | Total |
|---|---|---|---|---|---|---|---|
| | *N. meningitidis* | *N. gonorrhoeae* | *N. lactamica* | *N. polysaccharea* | *N. cinerea* | *N. bergeri* | |
| **123** | 1 | | | | | | **1** |
| **126** | 2 | | | | | | **2** |
| **129** | 2 | | | | | | **2** |
| **133** | 2 | | | | | | **2** |
| **134** | 1 | | | | | | **1** |
| **137** | 5 | | | | | | **5** |
| **139** | 1 | | | | | | **1** |
| **140** | 1 | | | | | | **1** |
| **143** | 1 | | | | | | **1** |
| **146** | | | | | 1 | | **1** |
| **147** | | | 1 | | | | **1** |
| **148** | 1 | | | | | | **1** |
| **149** | 3 | | | | | | **3** |
| **150** | | | | 1 | | | **1** |
| **151** | | 1 | | | | | **1** |
| **152** | | 1 | | | | | **1** |
| **155** | 1 | | | | | | **1** |
| **156** | 1 | | | | | | **1** |
| **158** | 2 | | | | | | **2** |
| **161** | 2 | | | | | | **2** |
| **169** | 1 | | | | | | **1** |
| **170** | | | 1 | | | | **1** |
| **175** | 1 | | | | | | **1** |
| **178** | 1 | | | | | | **1** |
| **184** | | | | | 1 | | **1** |
| **214** | 1 | | | | | | **1** |
| **228** | 1 | | | | | | **1** |
| **229** | 1 | | | | | | **1** |
| **232** | | | | 1 | | | **1** |
| **250** | 2 | | | | | | **2** |
| **252** | | 4 | | | | | **4** |
| **277** | 1 | | | | | | **1** |
| **282** | | | | 1 | | | **1** |
| **Total** | **9914** | **3767** | **140** | **17** | **4** | **1** | **13843** |
