# Supplementary material for: Neisseria gonorrhoeae employs two protein inhibitors to evade killing by human lysozyme
Source: PLoS Pathog. 2018 Jul 5;14(7):e1007080. doi: 10.1371/journal.ppat.1007080 (PMC6033460; doi:10.1371/journal.ppat.1007080)
Supplement: S3 Table — Clustal omega alignment of non-redundant ng1063 alleles (residues 80–110) from Neisseria identified in S2 Table. The Serine 83 and Lysine103 residues in red and blue, respectively, are conserved across all species. (PDF) [file ppat.1007080.s011.pdf]

S3 Table. Alignment of non-redundant NEIS1425 (*ng1063*) alleles from *Neisseria*.
Clustal omega alignment of non-redundant *ng1063* alleles (residues 80-110) from *Neisseria* identified in Supplemental Table 2. The Serine 83 and Lysine103 residues in red and blue, respectively, are conserved across all species.

Non-redundant Allele	Residues 80-110
92	VAASGERYTAENGLFGSGTEWHQKGGEAFFG
91	VAASGERYTAENGLFGSGTEWHQKGGEAFFG
149	VAASGERYTAEHGLFGNATEWHQKGGEAFFG
150	VAASGERYTAEHGLFGNGTEWHQKGGEAFFG
34	VAASGERYTAEHGLFGSGTEWHQKGGEAFFG
252	VAASGERYTAEHGLFGNGTEWHQKGGEAFFG
152	VAASGERYTAEHGLFGNGTEWHQKGGEAFFG
151	VAASGERYTAEHGLFGNGTEWHQKGGEAFFG
117	VAASGERYTAEHGLFGNGTEWHQKGGEAFFG
29	VAASGERYTAEHGLFGNGTEWHQKGGEAFFG
50	VAASGERYTAEHGLFGNGTEWHQKGGEAFFG
33	VAASGERYTAEHGLFGSGTEWHQKGGEAFFG
178	VAASGERYTAEHGLFGNGTEWHQKGGEAFFG
134	VAASGERYTAEHGLFGNGTEWHQKGGEAFFG
148	VAASGERYTAEHGLFGNGTEWHQKGGEAFFG
62	VAASGERYTAEHGLFGNGTEWHQKGGEAFFG
60	VAASGERYTAEHGLFGNGTEWHQKGGEAFFG
55	VAASGERYTAEHGLFGNGTEWHQKGGEAFFG
88	VAASGERYTAEHGLFGNGTEWHQKGGEAFFG
19	VAASGERYTAEHGLFGNATEWHQKGGEAFFG
90	VAASGERYTAEHGLFGNGTEWHQKGGEAFFG
6	VAASGERYTAEHGLFGNGTEWHQKGGEAFFG
8	VAASGERYTAEHGLFGNGTEWHQKGGEAFFG
228	VAASGERYTAEHGLFGNATEWHQKGGEAFFG
155	VAASGERYTAEHGLFGNATEWHQKGGEAFFG
15	VAASGERYTAEHGLFGNATEWHQKGGEAFFG
137	VAASGERYTAEHGLFGNGTEWHQKGGEAFFG
143	VAASGERYTAEHGLFGNATEWHQKGGEAFFG
140	VAASGERYTAEHGLFGNATEWHQKGGEAFFG
133	VAASGERYTAEHGLFGNATEWHQKGGEAFFG
53	VAASGERYTAEHGLFGNATEWHQKGGEAFFG
12	VAASGERYTAEHGLFGNATEWHQKGGEAFFG
120	VAASGERYTAEHGLFGNGTEWHQKGGEAFFG
250	VAASGERYTAEHGLFGNATEWHQKGGEAFFG
214	VAASGERYTAEHGLFGNATEWHQKGGEAFFG
161	VAASGERYTAEHGLFGNATEWHQKGGEAFFG
129	VAASGERYTAEHGLFGNATEWHQKGGEAFFG
74	VAASGERYTAEHGLFGNATEWHQKGGEAFFG
72	VAASGERYTAEHGLFGNATEWHQKGGEAFFG
42	VAASGERYTAEHGLFGNATEWHQKGGEAFFG
40	VAASGERYTAEHGLFGNATEWHQKGGEAFFG
30	VAASGERYTAEHGLFGNGTEWHQKGGEAFFG
18	VAASGERYTAEHGLFGNGTEWHQKGGEAFFG
14	VAASGERYTAEHGLFGNATEWHQKGGEAFFG
4	VAASGERYTAEHGLFGNATEWHQKGGEAFFG
11	VAASGERYTAEHGLFGNATEWHQKGGEAFFG
103	VAASGERYTAEHGLFGNGTEWHQKGGEAFFG
39	VAASGERYTAEHGLFGNGTEWHQKGGEAFFG
64	VAASGERYTAEHGLFGNATEWHQKGGEAFFG
175	VAASGERYTAEHGLFGNGTEWHQKGGEAFFG
80	VAASGERYTAEHGLFGNATEWHQKGGEAFFG
22	VAASGERYTAEHGLFGNGTEWHQKGGEAFFG
123	VAASGERYTAEHGLFGNATEWHQKGGEAFFG
158	VAASGERYTAEHGLFGNGTEWHQKGGEAFFG
277	VAASGERYTAEHGLFGNATEWHQKGGEAFFG
282	VAASGERYTAEHGLFGNATEWHQKGGEAFFG
104	VAASGERYTAEHGLFGNATEWHQKGGEAFFG
156	VAASGERYTAEHGLFGNATEWHQKGGEAFFG
139	VAASGERYTAEHGLFGNATEWHQKGGEAFFG
84	VAASGERYTAEHGLFGNATEWHQKGGEAFFG
82	VAASGERYTAEHGLFGNATEWHQKGGEAFFG
73	VAASGERYTAEHGLFGNATEWHQKGGEAFFG
61	VAASGERYTAEHGLFGNATEWHQKGGEAFFG
16	VAASGERYTAEHGLFGNATEWHQKGGEAFFG
2	VAASGERYTAEHGLFGNATEWHQKGGEAFFG
13	VAASGERYTAEHGLFGNATEWHQKGGEAFFG
126	VAASGERYTAEHGLFGNATEWHQKGGEAFFG
83	VAASGERYTAEHGLFGNGTEWHQKGGEAFFG
7	VAASGERYTAEHGLFGNGTEWHQKGGEAFFG
31	VAASGERYTAEHGLFGNGTEWHQKGGEAFFG
35	VAASGERYTAEHGLFGSGTEWHQKGGEAFFG
20	VAASGERYTAEHGLFGNGTEWHQKGSEAFFG
229	VAASGERYTAEHGLFGNATEWHQKGGEAFFG
25	VAASGERYTAEHGLFGNGTEWHQKGGEAFFG
43	VAASGERYTAEHGLFGNGTEWHQKGGEAFFG
49	VAASGERYTAEHGLFGNGTEWHQKGGEAFFG
184	VAASGERYTAEHGLFGNGTEWHQKGGEAFFG
32	VAASGERYTAEHGLFGNGTEWHQKGGEAFFG
47	VAASGERYTAEHGLFGNGTEWHQKGGEAFFG
108	VAASGERYTAEHGLFGNGTEWHQKGGEAFFG
79	VAASGERYTAEHGLFGNGTEWHQKGGEAFFG
232	VAASGERYTAEHGLFGNGTEWHQKGSEAFFG
169	VAASGEHYTAEHGLFGNGTEWHQKGGEAFFG
77	VAASGERYTAEHGLFGNGTEWHQKGGEAFFG
67	VAASGERYTAEHGLFGNATEWHQKGGEAFFG
65	VAASGERYTAEHGLFGNGTEWHQKGGEAFFG
41	VAASGERYTAEHGLFGNGTEWHQKGGEAFFG
1	VAASGERYTAEHGLFGNGTEWHQKGGEAFFG
119	VAASGERYTAEHGLFGNGTEWHQKGGEAFFG
170	VAASGERYTAEHGLFGNGTEWHQKGGEAFFG
37	VAASGERYTAEHGLFGNGTEWHQKGGEAFFG
147	VAASGERYTAEHGLFGNGTEWHQKGGEAFFG
36	VAASGERYTAEHGLFGNGTEWHQKGGEAFFG
146	VAASGERYTAEHGLFGNGTEWHQKGGEAFFG
78	VAASGERYTAEHGLFGNGTEWHQKGGEAFFG
Alignment	******:****:****..*******.*****
