# Supplementary material for: Neisseria gonorrhoeae employs two protein inhibitors to evade killing by human lysozyme
Source: PLoS Pathog. 2018 Jul 5;14(7):e1007080. doi: 10.1371/journal.ppat.1007080 (PMC6033460; doi:10.1371/journal.ppat.1007080)
Supplement: S4 Table — (PDF) [file ppat.1007080.s012.pdf]

S4 Table. Strains and plasmids used in this study.

Plasmid or Strains	Properties	Source or Reference
pKH35	Complementation vector, IPTG inducible, Cm^R	Hamilton, H.L. *et al.* (2005)
pMR68	Complementation vector, Anhydrotetracyclin inducible, Erm^R	Ramsey, M.E., *et al.* (2012)
pMR100	C-terminal 3XFLAG vector, Erm^R	Ramsey, M.E. *et al.* (2014)
pGEM-*Δ1981*	*1981* kanamycin insertion mutant, Kan^R	Humbert, M.V. *et al.* (2017)
pKH35_*1981*	*1981* complementation in pKH35, Cm^R	This work
pMR68_*1063*	*1063* complementation in pMR68, Erm^R	This work
pMR68_*1063*(S83A)	*1063*(S83A point mutation)-complementation in pMR68, Erm^R	This work
pMR68_*1063*(K103A)	*1063*(K103A point mutation)-complementation in pMR68, Erm^R	This work
pMR100_*1063*(WT)	*1063*(Wild-type)-3XFLAG construct in pMR100, Erm^R	This work
pMR100_*1063*(WT)_3'homology	*1063*(Wild-type)-3XFLAG construct with 3' 1063 homology in pMR100, Erm^R	This work
pMR100_*1063*(S83A)	*1063*(S83A point mutation)-3XFLAG construct in pMR100, Erm^R	This work
pMR100_*1063*(S83A)_3'homology	*1063*(S83A point mutation)-3XFLAG construct with 3' 1063 homology in pMR100, Erm^R	This work
pMR100_*1063*(K103A)	*1063*(K103A point mutation)-3XFLAG construct in pMR100, Erm^R	This work
pMR100_*1063*(K103A)_3'homology	*1063*(K103A point mutation)-3XFLAG construct with 3' 1063 homology in pMR100, Erm^R	This work
pMR100_*1981*(WT)	*1981*(Wild-type)-3XFLAG construct in pMR100, Erm^R	This work
pMR100_*1981*(WT)_3'homology	*1981*(Wild-type)-3XFLAG construct with 3' 1981 homology in pMR100, Erm^R	This work
pMR100_1981(S76A)_3'homology	*1981*(S76A)-3XFLAG construct with 3' 1981 homology in pMR100, Erm^R	This work
pMR100_1981(K99A)_3'homology	*1981*(K99A)-3XFLAG construct with 3' 1981 homology in pMR100, Erm^R	This work
pKH35_*1063*(WT)_3'homology	*1063*(Wild-type)-3XFLAG complementation in pKH35, Cm^R	This work
pKH35_*1063*(S83A)_3'homology	*1063*(S83A point mutation)-3XFLAG complementation in pKH35, Cm^R	This work
pKH35_*1063*(K103A)_3'homology	*1063*(K103A point mutation)-3XFLAG complementation in pKH35, Cm^R	This work
pKH35_*1981*(WT)_3'homology	*1981*(Wild-type)-3XFLAG complementation in pKH35, Cm^R	This work
pKH35_*1981*(S76A)_3'homology	*1981*(S76A)-3XFLAG complementation in pKH35, Cm^R	This work
pKH35_*1981*(K99A)_3'homology	*1981*(K99A)-3XFLAG complementation in pKH35, Cm^R	This work
pET22b::*1981*(WT)	*1981*(Wild-type)-His tag construct for expression in *E. coli*	Humbert, M.V. *et al.* (2017)
pET22b::*1063*(WT)	*1063*(Wild-type)-His tag construct for expression in *E. coli*	This work
WT, MS11 VD300 $P+_{nv}$	Wild-type RecA+ MS11 *N. gonorrhoeae* with nonvariable VD300 pilin	Ragland, S.A. *et al.* (2017)
ΔltgAΔltgD	KH560, *ltgA ltgD* double mutation in MS11	Cloud-Hansen, K.A. *et al.* (2008)
ΔltgA::ltgA-FLAG	RS518, *ltgA*-FLAG complementation in *ΔltgA* MS11, IPTG inducible	Shaub, R.E. *et al.* (2016)
ΔltgD::ltgD-FLAG	RS520, *ltgD*-FLAG complementation in *ΔltgD* MS11, IPTG inducible	Shaub, R.E. *et al.* (2016)
Δ1063	*1063* mutation in VD300 MS11	This work
Δ1981	*1981* mutation in VD300 MS11 with pGEM-*Δ1981*	This work
Δ1981Δ1063	*1981* mutation in *Δ1063* with pGEM-*Δ1981*	This work
Δ1981::1981+	*1981* complementation in *Δ1981* with pKH35_*1981*	This work
Δ1981Δ1063::1063+	*1063* complementation in *Δ1981Δ1063* with pMR68_*1063*	This work
Δ1981Δ1063::1063(WT)-FLAG	*1063*(Wild-type)-FLAG complementation in *Δ1981Δ1063* with pKH35_*1063*(WT)_3'homology	This work
Δ1981Δ1063::1063(S83A)-FLAG	*1063*(S83A point mutation)-FLAG complementation in *Δ1981Δ1063* with pKH35_*1063*(S83A)_3'homology	This work
Δ1981Δ1063::1063(K103A)-FLAG	*1063*(K103A point mutation)-FLAG complementation in *Δ1981Δ1063* with pKH35_*1063*(K103A)_3'homology	This work
Δ1981Δ1063::1981(WT)-FLAG	*1981*(Wild-type)-FLAG complementation in *Δ1981Δ1063* with pKH35_*1981*(WT)_3'homology	This work
Δ1063::1063(WT)-FLAG	*1063*(Wild-type)-FLAG complementation in *Δ1063* with pKH35_*1063*(WT)_3'homology	This work
Δ1981::1981(WT)-FLAG	*1981*(Wild-type)-FLAG complementation in *Δ1981* with pKH35_*1981*(WT)_3'homology	This work
Δ1981::1981(S76A)-FLAG	*1981*(S76A)-FLAG complementation in *Δ1981* with pKH35_*1981*(WT)_3'homology	This work
Δ1981::1981(K99A)-FLAG	*1981*(K99A)-FLAG complementation in *Δ1981* with pKH35_*1981*(WT)_3'homology	This work
1063(WT)-FLAG native	*1063*(Wild-type)-FLAG at its native locus	This work
ΔltgAΔltgD::1063+	*1063* complementation in *ΔltgAΔltgD* with pMR68_*1063*	This work
ΔltgAΔltgD::1981+	*1981* complementation in *ΔltgAΔltgD* with pKH35_*1981*	This work
ΔltgAΔltgDΔ1063	*1063* mutation in *ΔltgAΔltgD*	This work
ΔltgAΔltgDΔ1981	*1981* mutation in *ΔltgAΔltgD* using pGEM-*Δ1981*	This work
ΔltgAΔltgDΔ1981Δ1063	*1981* mutation in *ΔltgAΔltgDΔ1063* using pGEM-*Δ1981*	This work
